# A network model of glymphatic flow under different experimentally-motivated parametric scenarios

**DOI:** 10.1016/j.isci.2022.104258

**Published:** 2022-04-14

**Authors:** Jeffrey Tithof, Kimberly A.S. Boster, Peter A.R. Bork, Maiken Nedergaard, John H. Thomas, Douglas H. Kelley

**Affiliations:** 1 Department of Mechanical Engineering, University of Rochester, 235 Hopeman Building, Rochester 14627, NY, USA; 2 Department of Mechanical Engineering, University of Minnesota, 111 Church St SE, Minneapolis 55455, MN, USA; 3 Center for Translational Neuromedicine, Faculty of Health and Medical Sciences, University of Copenhagen, Blegdamsvej 3B, 2200 Copenhagen N, Copenhagen, Denmark; 4 Center for Translational Neuromedicine, Department of Neurosurgery, University of Rochester Medical Center, 601 Elmwood Avenue, Rochester 14642, NY, USA

**Keywords:** Neuroscience, Systems neuroscience, In silico biology

## Abstract

Flow of cerebrospinal fluid (CSF) through perivascular spaces (PVSs) in the brain delivers nutrients, clears metabolic waste, and causes edema formation. Brain-wide imaging cannot resolve PVSs, and high-resolution methods cannot access deep tissue. However, theoretical models provide valuable insight. We model the CSF pathway as a network of hydraulic resistances, using published parameter values. A few parameters (permeability of PVSs and the parenchyma, and dimensions of PVSs and astrocyte endfoot gaps) have wide uncertainties, so we focus on the limits of their ranges by analyzing different parametric scenarios. We identify low-resistance PVSs and high-resistance parenchyma as the only scenario that satisfies three essential criteria: that the flow be driven by a small pressure drop, exhibit good CSF perfusion throughout the cortex, and exhibit a substantial increase in flow during sleep. Our results point to the most important parameters, such as astrocyte endfoot gap dimensions, to be measured in future experiments.

## Highlights

• We model the CSF pathway as a network of hydraulic resistances
• Predictions are bracketed by analyzing parametric scenarios for unknown parameters
• Low-resistance PVSs and high-resistance parenchyma produce realistic flows
• Astrocyte endfoot gap size is among the important parameters to be measured

## Graphical abstract

**Figure:**
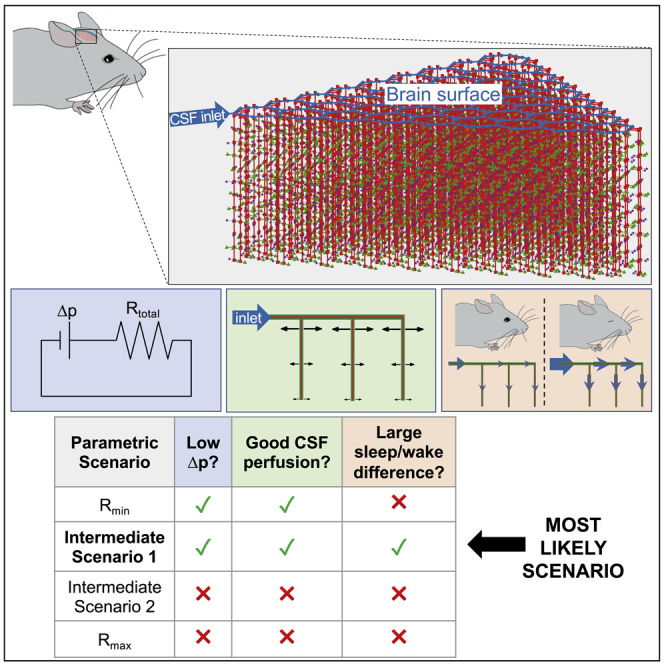

## Introduction

The brain lacks lymph vessels, so scientists have questioned whether a flow of cerebrospinal fluid (CSF) might play a pseudo-lymphatic role in transporting metabolic waste products (Milhorat, 1975). Early speculation was motivated by studies that found that tracers injected into the CSF were transported at rates faster than is possible by diffusion alone (Cserr et al., 1981; Rennels et al., 1985). Now, renewed interest has followed the *in vivo* observations of Iliff et al. (2012), who reported bulk flow of CSF through perivascular spaces (PVSs; annular channels around brain vasculature) of the murine brain, which aids clearance of amyloid-β, a peptide linked to Alzheimer’s disease; they named this clearance pathway the “glymphatic” (glial-lymphatic) system. Soon thereafter, Xie et al. (2013) demonstrated that this system is active primarily during sleep. Growing evidence suggests that glymphatic dysfunction may contribute to the progression of dementia (Nedergaard and Goldman, 2020) and worsened outcomes following stroke (Mestre et al., 2020), brain trauma (Sullan et al., 2018), and many other neurological disorders (Rasmussen et al., 2018).

The glymphatic pathway is hypothesized to consist of an influx of CSF along periarterial spaces which subsequently exchanges with extracellular fluid via bulk flow, facilitated by aquaporin-4 channels on the astrocyte endfeet lining the outer wall of PVSs, followed by an efflux along perivenous spaces and nerve sheaths (Plog and Nedergaard, 2018). Recent studies in humans have confirmed many of the key features of the glymphatic hypothesis (Ringstad et al., 2018; Fultz et al., 2019; Eide et al., 2021). Several experimental methods have been used to probe various parts of the glymphatic system. Two-photon microscopy offers excellent temporal and spatial resolution for *in vivo* measurements, but typically requires invasive surgery to place a cranial window and is limited to regions near the surface of the brain (Iliff et al., 2012; Schain et al., 2017; Mestre et al., 2018b, 2020). Magnetic resonance imaging (MRI) provides noninvasive brain-wide measurements, but temporal and spatial resolution are orders of magnitude lower, rendering PVSs smaller than the spatial resolution (Ringstad et al., 2018; Fultz et al., 2019; Taoka and Naganawa, 2020). Although *ex vivo* analysis of brain tissue offers high resolution throughout the brain, recent studies have revealed abnormal CSF flow immediately following cardiac arrest (Ma et al., 2019; Du et al., 2021) and collapse of PVSs during tissue fixation (Mestre et al., 2018b), casting doubt on such measurements. Hence, there remains much uncertainty regarding the precise CSF flow pathway and transport rates, including glymphatic efflux routes. Resolving such details may lead to novel strategies for prevention, diagnosis, and treatment of neurological disorders (Rasmussen et al., 2018).

Numerical modeling offers a powerful tool in which governing equations and physical constraints can fill voids where experimental measurements are not feasible. Indeed, much insight into the glymphatic system has already resulted from such studies (see the review articles by Ray and Heys (2019); Thomas (2019); Benveniste et al. (2019); Martinac and Bilston (2020); Rasmussen et al. (2021)). Here we develop numerical models of CSF flow through a substantial portion of the glymphatic system and use this model to make predictions under different scenarios that account for uncertainties in important geometric and material parameters. Because a fully-resolved fluid-dynamic model is not computationally feasible, our approach employs a hydraulic network model, as in prior work (Asgari et al., 2015; Faghih and Sharp, 2018; Rey and Sarntinoranont, 2018; Vinje et al., 2020). We investigate whether most CSF flows through the parenchyma or PVSs surrounding precapillaries, which we model as parallel pathways. Our attention to precapillary PVSs is motivated by (1) early experimental evidence of tracer transport through capillary PVSs (Rennels et al., 1985), (2) recent characterization of molecular markers suggesting PVSs are continuous from arterioles to capillaries to veins (Hannocks et al., 2018), and (3) recent theoretical arguments that diffusive transport in the parenchyma coupled with advective transport in precapillary PVSs might provide an effective clearance mechanism (Thomas, 2019).

**Figure 1:**
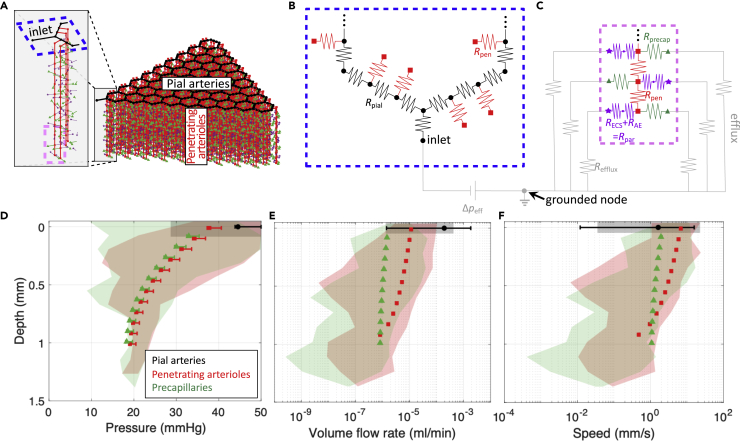
An idealized model of the cortical vasculature captures the salient features of blood flow, suggesting the vascular geometry used in our approach is reasonable (A) Diagram of the idealized vascular geometry, with colors indicating different vessel types. The blue and pink dashed lines show the regions that are enlarged in B-C. (B) Circuit schematic of the pial vasculature (black), which has several penetrating arterioles (red) branching from it. (C) Circuit schematic of a penetrating arteriole (red) which has a total of 11 precapillaries (green) branching from it (only three are shown). When we use a similar model to predict glymphatic CSF flow, we also include an equal number of parenchymal channels (purple). The gray circuit elements in B-C are not shown in A. (D–F) Pressure, volume flow rate, and speed for blood flow; in all three cases, the shaded regions indicate the range of values for a real vascular topology reported by Blinder et al. (2013), while the symbols and error bars indicate the mean and range of values, respectively, computed using the idealized geometry shown in panel A. See also Figure S1.

In order to improve on prior idealizations of the glymphatic pathway (Faghih and Sharp, 2018; Wang et al., 2021), we have developed a model of CSF flow in the murine brain based on measurements of the vascular connectivity performed by Blinder et al. (2010, 2013). We use the connectivity between different vessels in this model (Figures 1A–1C) to separately simulate either blood flow (for characterizing the influence of our idealized geometry) or CSF flow. The model includes flow associated with one of the major arteries branching from the circle of Willis, e.g., the middle cerebral artery (MCA), and thus includes flow in approximately one-fifth of the cortex. MRI studies (Iliff et al., 2013; Stanton et al., 2021) show that CSF enters pial PVSs at the circle of Willis, which is represented by the inlet node in our model, labeled in Fig. 1A–1B. The location of the outlet of our model (labeled “grounded node” in Figures 1B–1C) is ambiguous, as glymphatic efflux is not yet well-characterized; this could, for example, correspond to the subarachnoid space (SAS) or meningeal lymphatic vessels. The model geometry for the pial vasculature (Figure 1B) is based on a branching hexagonal model proposed by Blinder et al. (2010), with nine pial generations amounting to 45 hexagonal units and a total of 324 penetrating arterioles. This latter value approximately matches the number of penetrating arterioles in the vicinity of the MCA, 320, which we obtained by inspecting the pial arterial reconstructions available in the Supplemental Material of Adams et al. (2018). From data reported by Blinder et al. (2013), we determined that, on average, 11 precapillaries branch from each of the penetrating arterioles, which we assumed to be uniformly spaced (Figure 1C). Our hydraulic network model relates flow to the pressure differences that drive the flow and the hydraulic resistances that oppose the flow (pressure and resistance being analogous to voltage and electrical resistance in circuits). Note that this approach describes the time-averaged (net) volume flow rate and therefore neglects the oscillatory component of CSF flow, which is a reasonable approach since the Womersley number for PVS flow is small (Thomas, 2019). For blood flow (or CSF flow), the resistance through the capillary bed (or capillary PVSs) and venous circulation (or venous PVSs) is modeled using single parallel resistors, shown in gray in Figure 1C, with resistance $2.25\times 10^{7}$ mmHg⋅min/mL (or one mmHg⋅min/mL); see STAR Methods for details. Parenchymal flow (implemented only for CSF flow) is modeled using hydraulic resistances based on an analytical expression provided by Holter et al. (2017) (see STAR Methods). A full list of the parameters for the model is given in Table 1.

**Table 1:** Hydraulic network model parameters

Parameter	Value	Reference
Pial artery segment length	175 *μ*m	Blinder et al. (2010)
Pial artery diameter	46 *μ*m	Mestre et al. (2018b)
Penetrating arteriole length (*l*_pen_)	1000 *μ*m	Blinder et al. (2010)
Penetrating arteriole diameter (*d*_pen_)	11 *μ*m	Blinder et al. (2010)
Precapillary effective length	202 *μ*m	See STAR Methods
Precapillary diameter (*d*_precap_)	6 *μ*m	Miyawaki et al., 2020
Pial area ratio (Γ_pial_)	1.4	Mestre et al. (2018b)
Penetrating area ratio (Γ_pen_)	∗[0.36, 1.4]	
Precapillary area ratio (Γ_precap_)	∗[0.07, 0.36]	Yurchenco (2011), Reitsma et al., (2007)
Pial PVS permeability	N/A (open space)	Min Rivas et al. (2020)
Pen. & precap. permeability (*κ*_PVS_)	∗[4.5 × 10^−15^ m^2^, open]	Basser (1992)
Parenchymal permeability (*κ*_par_)	∗[1.2 × 10^−17^, 4.5 × 10^−15^] m^2^	Holter et al. (2017), Basser (1992)
Median arteriole-to-venule distance (*l*_a−v_)	128 *μ*m	Blinder et al. (2013)
Pial PVS shape	Optimal elliptical annulus	Tithof et al. (2019)
Penetrating PVS shape	Tangent eccentric annulus	Tithof et al. (2019)
Precapillary PVS shape	Concentric circular annulus	
Precapillaries per arteriole (*n*)	11	Blinder et al. (2013)
Dynamic viscosity	7 × 10^−4^Pa⋅s	
Endfoot wall thickness (*T*)	0.45 *μ*m	Mathiisen et al. (2010)
Endfoot gap width (*g*)	∗[20 nm, 5.1 *μ*m]	Mathiisen et al. (2010), Korogod et al. (2015)
Endfoot gap cavity fraction (*F*_*c*_)	∗[0.3%, 37%]	Mathiisen et al. (2010), Korogod et al. (2015)

Approximate bounds for uncertain variables, which are tested in this article, are indicated with an asterisk (∗).

## Results

### Characterizing the effects of network geometry idealization via blood flow simulations

In order to investigate whether the idealizations of our vascular model (e.g., hexagonal connectivity, homogeneity of pial artery diameter) significantly alter the distribution of flow, we compared blood flow in our model with blood flow predicted for the realistic network measured by Blinder et al. (2013). The idealized network was adjusted to cover an extent of vascular territory similar to that of the Blinder et al. study by matching the number of penetrating arterioles, resulting in a network with two pial generations (three hexagonal units), in contrast to the network shown in Figure 1A, which consists of nine pial generations or 45 hexagonal units. In Blinder et al. (2013), the authors measured the location and radius of all of the vessels within a section of the cortex, noting the connectivity between the vessels, and assigned a resistance to each segment based on a modified Hagen-Poiseuille law,

(Equation 1) $$R=\frac{32\mu L}{\pi r^{4}}\left[1-0.863e^{\frac{-r}{14.3\mu m}}+27.5e^{\frac{-r}{0.351\mu m}}\right],$$

where *r* is the vessel radius, *L* is the vessel length, *R* is the resistance of that segment of vessel, and μ is the dynamic viscosity of water. They then applied a constant pressure difference of 50 mmHg between the arterioles and venules at the surface of the cortex and solved for the flow in each vessel. The resulting ranges of pressures, volumetric flow rates, and velocities for one mouse are indicated by the shaded regions shown in Figures 1D–1F (see Figure S1 for results for two more mice). Based on Equation (1) and with a pressure difference of 50 mmHg between the inlet and outlet, we also predicted pressures, volume flow rates, and velocities for the idealized vascular geometry, which are plotted in Figures 1D–1F with solid symbols; the error bars indicate the range of values. The good agreement between the results for the realistic geometry (Blinder et al., 2013) and for the idealized geometry indicates that the idealization does not substantially alter the salient features of blood flow through the network. The smaller range of values observed for the idealized geometry is a result of the homogeneity of the idealization. These insights suggest that the idealized vascular geometry, which provides a framework for modeling glymphatic flow, is reasonable. Though it does not address the geometry of CSF circulation, we can infer that our results predicting glymphatic flow based on this idealized vascular geometry will likely also exhibit a narrower variation in pressure, volume flow rate, and flow speed than the actual network which has much greater heterogeneity.

### Dependence of glymphatic flow on permeability and PVS size

To model CSF flow through the glymphatic network, we enabled parenchymal flow (purple stars in Figures 1A and 1C), modeled three different types of PVSs – pial, penetrating, and precapillary – and assumed homogeneity in the shapes, sizes, and porosity of each of these different PVS types (see STAR Methods for a description of how the hydraulic resistance was computed for each pathway). Several variables needed to model fluid flow through the PVSs and parenchyma are unknown or have substantial uncertainty in their estimates. To overcome this challenge, we performed multiple simulations by bracketing the uncertain quantities (i.e., using the highest and lowest estimates of the uncertain quantities), based on a wide survey of the literature. We emphasize that in most cases, these bounds do not represent strict limits on feasible parameter ranges, but rather correspond to the extrema of values that have been reported in the literature or can be inferred from experimental data.

Bracketed parameters are indicated with an asterisk in Table 1. We considered four scenarios that lead to an overall resistance for the glymphatic network that is either maximal ($R_{\text{max}}$), minimal ($R_{\text{min}}$), or intermediate (Intermediate scenario 1, 2; i.e., a combination of one maximal and one minimal parameter set). For all these simulations, we matched the median pial PVS velocity to experimental measurements of 18.7 μm/s (Mestre et al., 2018b; Bedussi et al., 2017; Raghunandan et al., 2021); to obtain this match in flow speed, a different effective pressure drop $\text{Δ}p_{\text{eff}}$ was required for each different scenario. We modeled the pial PVSs as open (i.e., not porous) (Min Rivas et al., 2020) with a realistic, oblate shape (Tithof et al., 2019) and a PVS-to-artery cross-sectional area ratio of ${\text{Γ}}_{\text{pial}}=1.4$ (Mestre et al., 2018b). *In vivo* imaging studies suggest that pial PVSs are demarcated from the SAS (Mestre et al., 2018b; Schain et al., 2017), although some fluid may flow between the two compartments through stomata, which are pores up to a few microns in diameter (Pizzo et al., 2018); our model does not account for stomata. For penetrating PVSs, we used either an approximate upper bound on the area ratio ${\text{Γ}}_{\text{pial}}=1.4$ (i.e., that of pial PVSs) or an approximate lower bound on the area ratio ${\text{Γ}}_{\text{pial}}=0.36$ (i.e., the upper bound for the precapillary PVSs, discussed below). We modeled flow through the parenchyma, as well as porous penetrating and precapillary PVSs, using Darcy’s law; open (non-porous) penetrating PVSs were modeled as a tangent eccentric annulus (Tithof et al., 2019), and open precapillary PVSs were modeled using the analytical expression for flow through a concentric annulus (see STAR Methods).

**Figure 2:**
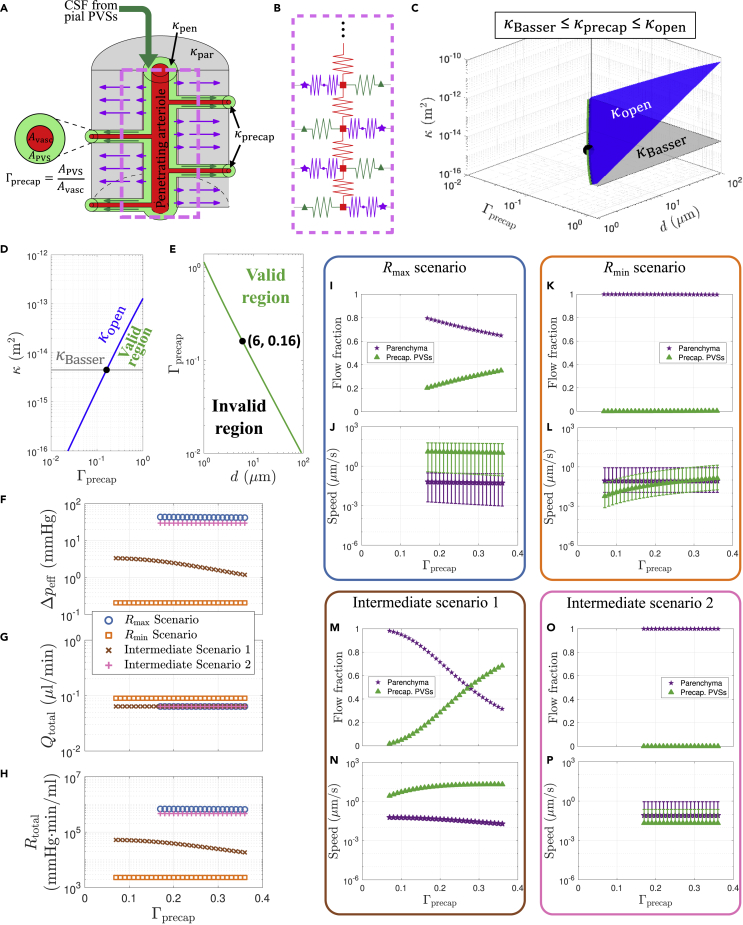
Simulations of CSF flow through the glymphatic network for different scenarios (A) Schematic illustrating the geometry of a penetrating PVS segment below the cortical surface (the same segment depicted in Figure 1C), with flow continuing through precapillary PVSs and/or the parenchyma. (B) Circuit schematic for the geometry shown in A (a greater portion of the network is shown in Figures 1B and 1C). Throughout this article, CSF flows through the precapillary PVSs or parenchyma are consistently plotted with green or purple arrows/symbols, respectively. (C–E) Plots indicating the range of feasible values of permeability based on measurements performed by Basser (1992) ($\kappa_{\text{Basser}}$) and the equivalent permeability for an open (non-porous) PVS ($\kappa_{\text{open}}$; see text). For $d_{\text{precap}}=6$μm, PVS sizes ${\text{Γ}}_{\text{precap}}<0.16$ are excluded for scenarios with $\kappa_{\text{PVS}}=\kappa_{\text{Basser}}$ ($R_{\text{max}}$ and Intermediate 2 scenarios). (F–H) The external pressure difference, total volumetric flow rate, and total hydraulic resistance for each of the four scenarios considered. (I–P) Flow fraction and flow speed through either precapillary PVSs or the parenchyma for the indicated scenarios. The symbols in panels J, L, N, and P indicate the mean flow speed across all space, while the error bars indicate the full range of values.

The four different scenarios we modeled arise from combining either the highest or lowest estimate of (1) the total parenchymal resistance and (2) the penetrating and precapillary PVS permeability, as detailed in Table 2. Minimum/maximum estimates of the total parenchymal resistance were obtained by lumping together the resistance from the gaps between astrocyte endfeet and the extracellular space (ECS; Figures 2A and 2B; see STAR Methods). Note that prior studies (Asgari et al., 2015; Jin et al., 2016; Wang et al., 2021) suggest CSF from penetrating PVSs primarily enters the ECS via gaps between astrocyte endfeet. We modeled flow through the gaps in the endfeet as flow between infinite parallel plates, with a fixed endfoot thickness of 0.45 μm based on measurements of the “intercellular cleft length” (Mathiisen et al., 2010) and variable width and cavity fraction for the gaps (Mathiisen et al., 2010; Korogod et al., 2015) (see Table 1 and Figure S2). The upper and lower bounds that we set on the parenchymal permeability $\kappa_{\text{par}}$ come from two commonly cited studies (Basser, 1992; Holter et al., 2017); multiple other studies (Morrison et al., 1994; Prabhu et al., 1998; Bobo et al., 1994; Neeves et al., 2006; Smith and Humphrey, 2007) have reported $\kappa_{\text{par}}$ values within these bounds. Basser (1992) performed experimental measurements that estimated $\kappa_{\text{par}}=4.5\times 10^{-15}$ m^2^. However, Holter et al. (2017) performed a numerical reconstruction of the neuropil, estimating $\kappa_{\text{par}}=1.2\times 10^{-17}$ m^2^, and speculated that the discrepancy with the earlier findings of Basser and other experimental studies may be because of fluid escaping to high-permeability pathways such as PVSs in those experiments. We therefore used this hypothesis as the basis for our $R_{\text{max}}$ scenario, with $\kappa_{\text{par}}=1.2\times 10^{-17}$ m^2^ and $\kappa_{\text{PVS}}=4.5\times 10^{-15}$ m^2^. For the $R_{\text{min}}$ scenario, we supposed that measurements of $\kappa_{\text{par}}=4.5\times 10^{-15}$ m^2^ from Basser (1992) accurately quantify the parenchymal permeability. To model flow through penetrating and precapillary PVSs with minimal resistance, we computed an effective permeability $\kappa_{\text{open}}$ that results from equating the volumetric flow rate predicted by Darcy’s law with the analytical expression for the volumetric flow rate for viscous flow through an open concentric circular annulus (see STAR Methods). This calculation defines a range of valid and invalid permeability values for a given PVS geometry, parameterized by the vessel diameter *d* and PVS-to-vessel area ratio Γ (Figures 2C–2E). We set $\kappa_{\text{PVS}}$ equal to the value of $\kappa_{\text{open}}$ for each corresponding geometry (penetrating and precapillary PVSs). Intermediate scenarios 1 and 2 come from choosing (1) $\kappa_{\text{par}}=1.2\times 10^{-17}$ m^2^ and $\kappa_{\text{PVS}}=\kappa_{\text{open}}$ or (2) $\kappa_{\text{par}}=\kappa_{\text{PVS}}=4.5\times 10^{-15}$ m^2^.

**Table 2:** The four different parametric scenarios tested in this article

	*κ*_par_ = 1*.*2 × 10^−17^ m^2^	*κ*_par_ = 4*.*5 × 10^−15^ m^2^
	*g* = 20 nm	*g* = 5*.*1 *μ*m
*κ*_PVS_ = 4*.*5 × 10^−15^ m^2^ Γ_pen_ = 0*.*36	*R*_max_	Intermediate 2
Open penetrating and precapillary PVSs Γ_pen_ = 1*.*4	Intermediate 1	*R*_min_

These four scenarios result from bracketing uncertain parameters related to: (left column) PVS permeability and penetrating PVS size and (top row) parenchymal permeability and astrocyte endfoot gap size

For each of the four scenarios we considered, we varied ${\text{Γ}}_{\text{precap}}$ (Figure 2A) from 0.07 to 0.36 (i.e., the precapillary PVS gap ranged from 0.1 to 0.5 μm). These values come from estimates of the size of a basement membrane (Yurchenco, 2011) or the endothelial glycocalyx (Reitsma et al., 2007), which are the respective smallest and largest anatomical structures likely to form the contiguous portion of the PVS network at the precapillary level. In general, the anatomical details of which spaces are contiguous with penetrating PVSs are not well-understood; for a more in-depth discussion of potential PVS routes at the level of microvessels, see Hladky and Barrand (2018). For the $R_{\text{min}}$ and Intermediate 2 scenarios (with $\kappa_{\text{PVS}}=\kappa_{\text{Basser}}=4.5\times 10^{-15}$ m^2^), we found that an open precapillary PVS would result in an effective permeability $\kappa_{\text{open}}<\kappa_{\text{Basser}}$ for ${\text{Γ}}_{\text{precap}}<0.16$ (Figure 2E). By definition, $\kappa_{\text{open}}$ provides the upper limit on permeability, and because these two scenarios assume $\kappa_{\text{Basser}}$ provides the lower limit of PVS permeability, we exclude ${\text{Γ}}_{\text{precap}}<0.16$ (i.e., precapillary PVS gap widths below 0.23 μm) from further analysis in these two scenarios.

The effective pressure drop $\text{Δ}p_{\text{eff}}$ required to drive flow through the glymphatic network is plotted in Figure 2F for all four scenarios. By “effective” pressure drop, we mean that we have driven flow through the network using a single pressure source (Figure 1B), but the actual pressure gradients driving glymphatic flow – the source of which is actively debated – may be much more complex. The effective pressure drop may be thought of as $Q_{\text{total}}/R_{\text{total}}$, where $Q_{\text{total}}$ and $R_{\text{total}}$ are the total volume flow rate and resistance for the entire glymphatic network, respectively, even if an external pressure drop of that magnitude does not exist. Potential sources of the pressure gradients that drive the observed flows include arterial pulsations (Mestre et al., 2018b), functional hyperemia (Kedarasetti et al., 2020), and osmotic effects (Plog et al., 2018; Halnes et al., 2019; Rasmussen et al., 2021). The largest effective pressure drop (43 mmHg) is required for the $R_{\text{max}}$ case with ${\text{Γ}}_{\text{precap}}=0.17$, whereas the $R_{\text{min}}$ case requires a drop of only 0.21 mmHg (which does not vary appreciably with ${\text{Γ}}_{\text{precap}}$). CSF pressure gradients have never been measured in mice, but a prior study (Penn and Linninger, 2009) numerically estimated that the largest feasible transmantle pressure difference in humans is approximately 1 mmHg, and hence the $R_{\text{min}}$ scenario is possible and Intermediate Scenario 1 is of marginal feasibility, whereas the $R_{\text{max}}$ and Intermediate 2 scenarios are very unlikely. The total volumetric flow rate through the entire network (Figure 2G), which is approximately one-fifth of the full cortical glymphatic network, varies from 0.063 to 0.089 μL/min for all cases considered here; this relatively narrow range of values is a consequence of our requirement that the median flow speed in the pial PVSs match experimental measurements (Mestre et al., 2018b). With negligible variation in $Q_{\text{total}}$ for a given scenario, the total hydraulic resistance of the network (Figure 2H) is linearly proportional to the effective pressure drop, resulting in a similar functional dependence for each scenario.

**Figure 3:**
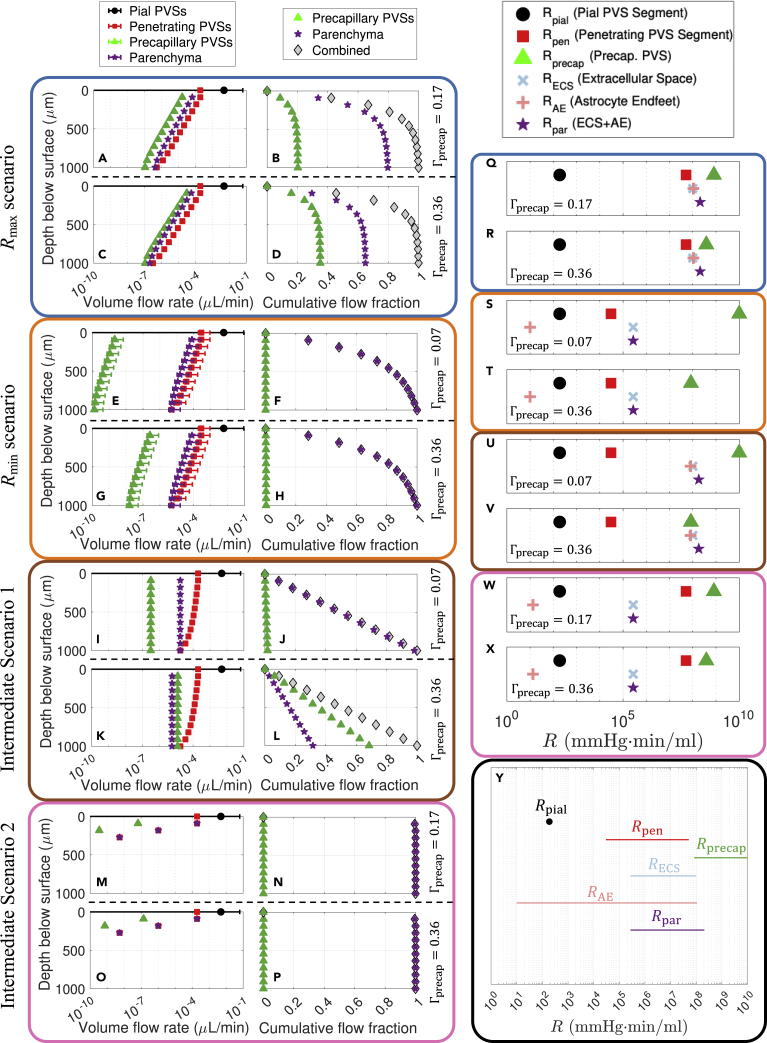
Cortical CSF perfusion in different scenarios (A–P) (A, C, E, G, I, K, M, O) The volume flow rate across the depth of the cortex, and (B, D, F, H, J, L, N, P) the cumulative flow fraction, defined as the fraction of total volume perfused from the surface of the brain to a given depth of the cortex, for the different indicated scenarios. The legends at the top apply to each corresponding column of plots. Note that panels E−F and I−J have small precapillary PVSs (${\text{Γ}}_{\text{precap}}=0.07$), whereas panels C−D, G-H, K−L, and O−P have large precapillary PVSs (${\text{Γ}}_{\text{precap}}=0.36$). Panels A−B and M−N have precapillary PVSs of intermediate sizes (${\text{Γ}}_{\text{precap}}=0.17$) which satisfy $\kappa_{\text{open}}\geq \kappa_{\text{Basser}}$. The symbols indicate mean values while the error bars indicate the full range of values. (Q–X) Plots indicating the hydraulic resistance for a single segment of the network in each scenario, as indicated by the color of the bounding box and the ${\text{Γ}}_{\text{precap}}$ label. (Y) A plot of the ranges of hydraulic resistance considered across different scenarios in this study for each individual resistive element. See also Figures S3, S4, S5.

**Figure 4:**
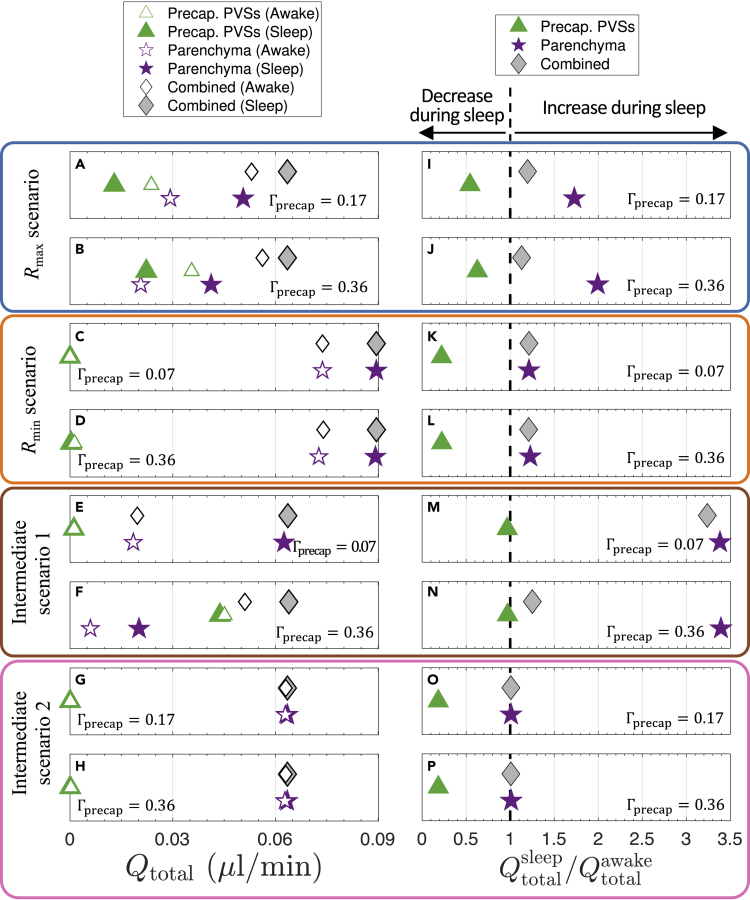
Modeled glymphatic flow in wakefulness and sleep (A–H) Volumetric flow rate $Q_{\text{total}}$ summed over the entire network for different routes during either sleep or wakefulness, as indicated by the legend at the top; four different scenarios are considered, each with either small or large precapillary PVSs, as indicated. (I–P) The factor by which flow through precapillary PVSs, parenchyma, or both routes combined changes during sleep compared to wakefulness, quantified as $Q_{\text{total}}^{\text{sleep}}/Q_{\text{total}}^{\text{awake}}$, for the different indicated scenarios. The black dashed line corresponds to a value of 1, indicating no change; values to the right or left of this line correspond to an increase or a decrease, respectively, in the indicated volumetric flow rate during sleep. Note the different limiting precapillary PVS sizes ${\text{Γ}}_{\text{precap}}$ indicated in the corner of each panel. See also Figure S6.

We next investigated the percentage of flow that passes through the parenchyma versus the precapillary PVSs and the associated flow speed for each case (Figures 2I–2P). We found that when $\kappa_{\text{par}}=1.2\times 10^{-17}$ m^2^ ($R_{\text{max}}$ and Intermediate 1 scenarios) there is a comparable fraction of total flow through the parenchyma and precapillary PVSs (Figures 2I and 2M). However, if $\kappa_{\text{par}}=4.5\times 10^{-15}$ m^2^ ($R_{\text{min}}$ and Intermediate 2 scenarios), virtually all of the flow passes through the parenchyma with a negligible amount passing through the precapillary PVSs (Figures 2K and 2O). Consequently, only the former two cases show a substantial dependence on ${\text{Γ}}_{\text{precap}}$, with the percentage of flow through precapillary PVSs varying from 20% to 35% as ${\text{Γ}}_{\text{precap}}$ is varied from 0.17 to 0.36 for the $R_{\text{max}}$ scenario, or from 1.8% to 68% as ${\text{Γ}}_{\text{precap}}$ is varied from 0.07 to 0.36 for Intermediate scenario 1. The average flow speeds are plotted in Figures 2J, 2L, 2N, and 2P, with error bars indicating the full range of the data. The mean values for the flow speed through the parenchyma are quite similar for all four scenarios, with the average speed varying from 0.053 μm/s to 0.065 μm/s for the $R_{\text{max}}$ scenario, or from 0.060 μm/s to 0.019 μm/s for Intermediate scenario 1, as ${\text{Γ}}_{\text{precap}}$ is increased. For the $R_{\text{min}}$ scenario (Intermediate scenario 2), the mean speed is 0.086 (0.081) μm/s and does not vary appreciably with ${\text{Γ}}_{\text{precap}}$. We caution that the plotted parenchymal flow speeds are not mean values across the parenchyma; they are computed at the outer wall of the PVS, so they should be interpreted as upper bounds on the parenchymal flow speed, which varies spatially. The mean precapillary flow speeds, in contrast to parenchymal speeds, show substantial variation throughout the four scenarios. The average speed varies from 13 μm/s to 10 μm/s for the $R_{\text{max}}$ scenario, or from 2.7 μm/s to 20 μm/s for Intermediate scenario 1, as ${\text{Γ}}_{\text{precap}}$ is increased. For the $R_{\text{min}}$ scenario, the mean speed varies from 0.0058 to 0.13 μm/s as ${\text{Γ}}_{\text{precap}}$ is increased, but for Intermediate scenario two the mean speed is 0.021 μm/s and does not vary with ${\text{Γ}}_{\text{precap}}$. Figures S3 and Figure 1, Figure 2, Figure 3, Figure 4 show how the speed and pressure vary throughout the network for the four scenarios each with maximum or minimum ${\text{Γ}}_{\text{precap}}$.

### Quantifying CSF perfusion of tissue for different scenarios

Numerous studies in both humans and mice have reported that tracers injected into CSF penetrate below the brain’s surface over relatively short time scales (Gaberel et al., 2014; Ringstad et al., 2018; Eide et al., 2021; Taoka and Naganawa, 2020). Furthermore, there is growing evidence that CSF flow through the glymphatic pathway is important for the removal of metabolic waste (Koundal et al., 2020; Gu et al., 2020; Eide et al., 2021), including amyloid-β (Iliff et al., 2012; Xie et al., 2013; Roberts et al., 2014; Shokri-Kojori et al., 2018), which is produced throughout the brain. Hence, one may reasonably expect a uniform perfusion of CSF throughout the depth of the cortex to explain observations in tracer experiments and the physiological necessity of adequate waste removal. Consequently, we next computed the volume flow rate through pial PVSs, penetrating PVSs, precapillary PVSs, and the parenchyma for each of eight cases (the four scenarios introduced previously, each with either the maximum or minimum value of ${\text{Γ}}_{\text{precap}}$), as shown in the left columns of each scenario in Figure 3. It is immediately clear that when penetrating PVS resistance is minimal ($R_{\text{min}}$ and Intermediate 1 scenarios), a significant volume of CSF penetrates into the deep cortex (Figures 3E, 3G, 3I, and 3K). However, if penetrating PVS resistance is high ($R_{\text{max}}$ and Intermediate 2 scenarios), the volume flow rate drops off more rapidly with depth (Figures 3A, 3C, 3M, and 3O). Figure S5 provides a visualization of how the volume flow rate varies throughout the network.

To characterize the CSF perfusion, we plotted the cumulative flow fraction (i.e., the fraction of the total volume flow rate perfused from the surface of the brain to a given depth) in the right columns for each scenario in Figure 3. The $R_{\text{max}}$ scenario has fairly poor CSF perfusion, with 81% (${\text{Γ}}_{\text{precap}}=0.17$) to 84% (${\text{Γ}}_{\text{precap}}=0.36$) of the total CSF exiting each penetrating PVS within 270 μm of the surface. Comparing the flows for small versus large precapillary PVSs (Figures 3B and 3D), it is clear that more flow reroutes through the PVSs in the latter case, consistent with Figure 2I. Among all cases, Intermediate scenario 2 exhibits the worst CSF perfusion, with 100% of the total CSF perfusing within 270 μm of the surface (Figures 3N and 3P); this scenario exhibits negligible dependence on ${\text{Γ}}_{\text{precap}}$. In contrast, the $R_{\text{min}}$ scenario exhibits moderately uniform CSF perfusion, with 63% of the total CSF perfusing within 270 μm of the surface (Figures 3F and 3H); this scenario also exhibits weak dependence on ${\text{Γ}}_{\text{precap}}$. By far the best CSF perfusion is observed in Intermediate scenario 1, for which 27 and 28% of the CSF is perfused within 270 μm of the surface for ${\text{Γ}}_{\text{precap}}=0.07$ and ${\text{Γ}}_{\text{precap}}=0.36$, respectively (Figures 3J and 3L; perfectly uniform CSF perfusion corresponds to 27% at 270 μm). Although the total CSF perfusion remains approximately constant for different precapillary PVS sizes, as ${\text{Γ}}_{\text{precap}}$ is increased a greater fraction of the flow reroutes from the parenchyma to the precapillary PVSs (compare Figures 3I–3J with 3K–3L), consistent with the flow fractions plotted in Figure 2M.

The variations in CSF perfusion through the depth of the cortex for these different scenarios can be understood by comparing the hydraulic resistance of individual segments of the network, as shown in Figures 3Q–3X; several of these values are also provided in Table S1. When $R_{\text{pen}}$ is substantially smaller than both $R_{\text{par}}$ and $R_{\text{precap}}$ (Intermediate scenario 1; Figures 3U–3V), excellent, uniform CSF perfusion occurs (Figures 3J and 3L). However, a lesser separation in resistance values ($R_{\text{max}}$ and $R_{\text{min}}$ scenarios; Figures 3Q–3T) leads to less uniformity in the CSF perfusion (Figures 3B, 3D, 3F, and 3H). When $R_{\text{pen}}$ is much greater than $R_{\text{par}}$ (Intermediate scenario 2; Figures 3W–3X), virtually all fluid exits through the parenchymal nodes closest to the surface of the brain and CSF perfusion is negligible at deeper nodes (Figures 3N and 3P). The relative flow through the parenchyma versus precapillary PVSs can also be understood by comparing $R_{\text{precap}}$ and $R_{\text{par}}$. For cases where there is substantial CSF perfusion, if the value of $R_{\text{precap}}$ and $R_{\text{par}}$ are comparable (Figures 3Q–3R and 3V), then a comparable fraction of fluid will flow through each route (Figures 3B, 3D, and 3L), with greater flow through the path of lower resistance. Two additional points are notable. The value of $R_{\text{pial}}$ is much less than $R_{\text{pen}}$ in every scenario, which ensures uniform perfusion of CSF across the pial PVS network (i.e., an approximately equal amount of CSF flows through both a distal penetrating PVSs and a proximal one). Also, the uncertainties in the cavity fraction and gap width of the astrocyte endfeet lead to a huge range in possible values of $R_{\text{AE}}$ (Figure 3Y). In the $R_{\text{max}}$ and Intermediate 1 scenarios, the astrocyte endfeet constitute a significant barrier to flow entering the parenchyma (Figures 3Q–R and U–V); however, in the $R_{\text{min}}$ and Intermediate 2 scenarios (Figures 3S–3T and 3W–3X), $R_{\text{AE}}$ is very small and hence plays a negligible role in determining CSF flow through the parenchyma.

### Glymphatic flow during wakefulness versus sleep

We carried out additional calculations with our model aimed at investigating the increase in tracer influx during sleep/anesthesia reported by several studies (Xie et al., 2013; Plog et al., 2018; Hablitz et al., 2019, 2020). The CSF simulations presented up to this point (Figures 2 and 3) correspond to sleep conditions (or, comparably, conditions under ketamine-xylazine anesthesia). To model the change in flow during wakefulness, we used the Kozeny-Carman equation (see STAR Methods) to estimate that $\kappa_{\text{par}}$ decreases by a factor of about 5.5 in wakefulness, compared to sleep. We repeated the simulations of the eight scenarios presented in Figure 3 using a parenchymal permeability that was 5.5 times smaller, but all other parameters (including the imposed pressure drop) were left unchanged for each scenario. We then compared these results to the results from each corresponding simulation under sleep conditions. The total volume flow rates through the entire model network for wake and sleep in each of the eight scenarios are plotted in Figures 4A–4H. The combined flow for awake conditions (open gray diamonds) varies substantially across different scenarios, whereas for sleep (filled gray diamonds) all correspond to $Q_{\text{total}}$ of either 0.063 or 0.089 μL/min, consistent with Figure 2G.

We quantified the sleep/wake change in flow by plotting the ratio of volume flow rates $Q_{\text{total}}^{\text{sleep}}/Q_{\text{total}}^{\text{awake}}$, shown in Figures 4I–4P. For sleep compared to wakefulness, in every scenario the volume flow rate decreases for precapillary PVSs and increases for the parenchyma, leading to an overall increase in the combined volume flow rate. This is expected because the increased parenchymal permeability during sleep leads to an overall reduction in the hydraulic resistance of the network, and locally this change will reroute some precapillary PVS flow through the parenchyma. We find that the wake/sleep increase in combined volume flow rate is largest for small precapillary PVSs (Figures 4I, 4K, 4M, and 4O); this combined increase is small for Intermediate scenario 2 (0.8%), $R_{\text{max}}$ (13%–20%), and $R_{\text{min}}$ (21%), but up to 220% for Intermediate scenario 1. In this latter scenario, however, there is substantial sensitivity to the size of the precapillary PVSs (Figures 4E–4F and 4M−4N), which arises because parenchymal flow dominates the combined transport for the ${\text{Γ}}_{\text{precap}}=0.07$ case (and is therefore sensitive to wake/sleep changes in $\kappa_{\text{par}}$), whereas precapillary PVS flow dominates the combined transport for the ${\text{Γ}}_{\text{precap}}=0.36$ case (and is therefore insensitive to wake/sleep changes in $\kappa_{\text{par}}$); this observation is consistent with Figure 2M. Of all of our wake/sleep simulations, none exhibits an increase in combined flow greater than 220%.

## Discussion

In this study, we have developed a numerical model of a substantial portion of the glymphatic system in the murine brain. This model is based on an idealized vascular geometry inspired by detailed measurements reported by Blinder et al. (2010, 2013), and we characterized the effects of idealizing the vascular geometry by first performing simulations of blood flow (Figure 1). In modeling CSF flow through the glymphatic pathway, we matched median pial CSF velocity to experiments (Mestre et al., 2018b), we realistically modeled pial PVSs as open (non-porous) (Min Rivas et al., 2020) and oblate (Tithof et al., 2019), and we used experimentally measured mean vessel diameters and lengths. To overcome the multiple uncertainties in other parameters, we set reasonable bounds (Table 1) and performed several simulations corresponding to different combinations of the extreme values of the uncertain parameters (Figure 2). It should be noted that these bounds are not strict extrema, but rather correspond to maximum/minimum values of each quantity as reported in various experimental studies. This “bracketing” approach included upper and lower bounds on the hydraulic resistance for penetrating PVSs, precapillary PVSs, and the parenchyma (based on a lumped model of astrocyte endfeet and the parenchymal ECS). Our model assumes CSF passes from penetrating PVSs to either precapillary PVSs or through the parenchymal ECS via a paracellular route through gaps between astrocyte endfeet (Rasmussen et al., 2021). Ultimately, our goal was to investigate different scenarios to test which parameter regimes are feasible and explain as much experimental data as possible. We focused primarily on quantifying the required pressure drops, flow fraction and speed, cortical CSF perfusion, and sleep/wake changes in volumetric flow rate.

The pressure drops and total volumetric flow rates we computed (Figures 2F–2G) provide novel insights. The two scenarios with high penetrating PVS resistance $R_{\text{pen}}$ ($R_{\text{max}}$ and Intermediate scenario 2) require infeasibly large pressure drops between 30 and 43 mmHg. This renders both scenarios very unlikely because such a large pressure drop is even greater than the typical systolic-diastolic variation in blood pressure of about 20 mmHg (Mattson, 2001), which is thought to provide an absolute upper bound for the pressure drop driving glymphatic flow (Faghih and Sharp, 2018). The $R_{\text{min}}$ scenario, however, requires the lowest pressure drop by definition, which is only 0.21 mmHg. Such a pressure drop is feasible and in line with estimates for the transmantle pressure difference (Penn and Linninger, 2009) (i.e., that between the SAS and lateral ventricles); note however that Penn and Linninger (2009) is a model of human anatomy. For Intermediate scenario 1, the required pressure drop is moderately larger, varying from 1.2 to 3.3 mmHg for ${\text{Γ}}_{\text{precap}}$ from 0.36 to 0.07, respectively. Such a pressure drop is perhaps of marginal feasibility, possibly requiring driving mechanism(s) beyond simply a transmantle pressure difference (additional mechanisms are discussed further below).

Because we matched the median pial CSF velocity to experimental measurements (Mestre et al., 2018b), we find $Q_{\text{total}}=0.064$μL/min for every scenario, except the $R_{\text{min}}$ scenario in which$Q_{\text{total}}=0.089$ (Figure 2G). The reason $Q_{\text{total}}$ is moderately larger in the $R_{\text{min}}$ scenario is because the minimal resistances of penetrating PVSs and parenchyma (Figures 3S–3T) allow more fluid to exit the network along the parenchymal nodes most proximal to the inlet, which is perhaps discernible in Figure S5B. Our model represents approximately one-fifth of the cortical glymphatic network (e.g., in the vicinity of one MCA), so the total CSF volume flow rate through cortical PVSs would be approximately 0.32 μL/min, much larger than the CSF production rate of the choroid plexus, which has recently been measured to be about 0.1 μL/min for young, healthy, anesthetized mice (Liu et al., 2020). Although this measurement involves invasive techniques, Karimy et al. (2015) (who developed the technique used by Liu et al. (2020) in rats) reported that results were consistent with a prior method; still, this measurement may be an underestimate, as the approach excludes CSF production at the 4th ventricle.

Multiple potential explanations exist for the discrepancy between estimates of CSF production and the larger volume flow rate from our model, some of which depend on the details of pial PVSs. The pial PVSs that we have modeled are extensions of the SAS, and prior studies have suggested that not all fluid in pial PVSs continues to penetrating PVSs but rather a portion of the flow continues directly from PVSs of pial arteries to those of veins (Bedussi et al., 2017; Pizzo et al., 2018; Ma et al., 2019). Furthermore, not all CSF from the SAS enters pial PVSs; Lee et al. (2018) delivered a tracer to the cisterna magna in rats and determined that approximately 20% reached the parenchyma, with the rest following CSF efflux routes, including the arachnoid villi, cribriform plate, and cranial and spinal nerves. Hence, it is likely that only a fraction of the total CSF enters pial PVSs, and perhaps not all CSF in pial PVSs continues through penetrating PVSs and into the parenchyma. We note that our model does not include direct flow from the SAS into penetrating PVSs (Pizzo et al., 2018). Our prediction of a volume flow rate *larger* than CSF production thus suggests that either (1) published *in vivo* measurements of fluid velocities (Mestre et al., 2018b; Bedussi et al., 2017) are inaccurately large, (2) the pial geometry in our model is too idealized and greatly overestimates the volume flow rate, (3) published measurements of CSF production rates are inaccurately small, and/or (4) the fraction of CSF in pial PVSs which does not enter penetrating PVSs is able to flow back into the SAS and reenter pial PVSs of arteries, forming a kind of recirculation along the surface of the brain. Future studies could test these possibilities. Option (1) seems unlikely because of the good agreement between two independent studies (17 μm/s versus 18.7 μm/s reported by Bedussi et al. (2017) and Mestre et al. (2018b), respectively). Option (2) perhaps plays a role, and future numerical studies with improved modeling of the pial PVS geometry should investigate this possibility. It is likely that option (3) might contribute to the discrepancy, but such experiments are challenging and always have confounding factors. Option (4) may contribute as well; future particle tracking experiments should investigate possibilities (1) and (4).

The values of hydraulic resistance computed with our model can be directly compared to those of prior work. Faghih and Sharp (2018) developed a network model of flow through periarterial spaces and computed a total network resistance of 1.14 mmHg⋅min/mL. This value is about 2000 times lower than the lowest hydraulic resistance we compute, R=2300 mmHg⋅min/mL for the $R_{\text{min}}$ scenario. This discrepancy is probably primarily because Faghih and Sharp modeled glymphatic flow in a human, with far more parallel channels than we have considered. Vinje et al. (2020) developed a compartmental model to estimate how elevated intracranial pressure may affect CSF outflow pathways. Although their study modeled human anatomy, they used parameters similar to Intermediate scenario 1 in this study and reported that the hydraulic resistance of the parenchyma was comparable to that of the PVSs, which is in good agreement with our observations.

We find that a substantial fraction of the CSF flowing through penetrating PVSs continues through the parenchyma in every scenario, with values ranging from 32% (Intermediate scenario 1 with ${\text{Γ}}_{\text{precap}}=0.36$; Figure 2M) to 100% ($R_{\text{min}}$ and Intermediate 2 scenarios; Figures 2K and 2O). In fact, a greater portion of CSF flows through the parenchyma than precapillary PVSs in every scenario except Intermediate scenario 1 with large precapillary PVSs (${\text{Γ}}_{\text{precap}}\geq 0.27$). For the $R_{\text{max}}$ and Intermediate 1 scenarios, $\kappa_{\text{par}}\ll \kappa_{\text{PVS}}$ but in the penetrating PVSs the parenchymal-to-precapillary PVS surface area ratio is large (∼270), leading to comparable hydraulic resistance for these two parallel pathways (Figures 3Q–3R and 3V). The mean parenchymal flow speeds we find are surprisingly robust across different scenarios, with values ranging from 0.019 to 0.086 μm/s depending on the scenario and value of ${\text{Γ}}_{\text{precap}}$ (Figures 2J, 2L, 2N, and 2P). Our upper bound is in agreement with the lower bound of flow speeds, 0.083 μm/s, reported by Ray et al. (2019). In addition, our lower bound is in agreement with results from Holter et al. (2017), in which parenchymal flow speed near the outer wall of the PVS is about 0.035 μm/s (see Figure 3 in Holter et al. (2017)). For cases in which the precapillary PVS flow fraction is non-negligible (>0.5%; $R_{\text{max}}$ and Intermediate 1 scenarios), the speeds are also fairly robust, ranging from 2.7 to 20 μm/s (Figures 2J and 2N). This moderate insensitivity to precapillary PVS size (${\text{Γ}}_{\text{precap}}$) – especially for the $R_{\text{max}}$ scenario – can be understood as follows: as the cross-sectional area $A_{\text{PVS}}$ increases, the hydraulic resistance $R_{\text{precap}}$ decreases causing the volume flow rate *Q* to increase, rendering the flow speed ($=Q/A_{\text{PVS}}$) approximately constant. To the best of our knowledge, this is the first time precapillary PVS flow speed has been predicted.

We assessed whether each scenario exhibits uniformity in cortical CSF perfusion, which we expect based on reports of tracer penetration below the brain’s surface (Gaberel et al., 2014; Ringstad et al., 2018; Eide et al., 2021; Taoka and Naganawa, 2020) and evidence that flow is important for metabolic waste removal (Iliff et al., 2012; Xie et al., 2013; Roberts et al., 2014; Shokri-Kojori et al., 2018; Koundal et al., 2020; Gu et al., 2020; Eide et al., 2021). Our simulations revealed near-perfect cortical CSF perfusion for Intermediate scenario 1, moderately uniform CSF perfusion for the $R_{\text{min}}$ scenario, fairly poor CSF perfusion for the $R_{\text{max}}$ scenario, and negligible CSF perfusion below the brain surface for Intermediate scenario 2 (Figure 3). As discussed above, good uniformity in CSF perfusion can be understood as a consequence of scale separation in the hydraulic resistance of the three sequential CSF routes: pial PVSs, penetrating PVSs, and parenchyma/precapillary PVSs (Figures 3Q–3X). Poor CSF perfusion occurs if these resistances are comparable (Figures 3Q–3R) or do not increase in the aforementioned order (Figures 3W–3X). This observation provides an argument in favor of large parenchymal resistance, which could arise because of tight astrocyte endfeet gaps, a low-permeability parenchymal ECS, or a combination of the two. Furthermore, the separation in scale between pial and penetrating PVS resistance ensures that CSF is uniformly perfused from the pial PVSs to the penetrating PVSs. This need for separation in scale may explain why pial PVSs have an oblate shape that minimizes their hydraulic resistance (Tithof et al., 2019).

We performed simulations aimed at capturing the increase in CSF flow during sleep compared to wakefulness (Xie et al., 2013; Plog et al., 2018). Multiple studies demonstrate that glymphatic transport is enhanced under ketamine/xylazine (K/X) anesthesia, resembling natural sleep, and inhibited under isoflurane, resembling wakefulness (Xie et al., 2013; Plog et al., 2018; Hablitz et al., 2019; Stanton et al., 2021); indeed, both the prevalence of slow (delta) waves and the ECS porosity under K/X are comparable to natural sleep (Xie et al., 2013). These studies comparing K/X and isoflurane highlight the heterogeneity of tracer transport in different regions of the brain, often with two- to four-fold greater tracer influx under K/X, compared to isoflurane. We found that $R_{\text{min}}$, $R_{\text{max}}$, and Intermediate scenario 2 all exhibit less than a 22% increase in combined volume flow rate during sleep compared to wakefulness (Figures 4I–4L and 4O–4P); however, we found a 3.2-fold increase in combined volume flow rate for Intermediate scenario 1 with small precapillary PVSs (Figures 4M). Increased tracer transport can be estimated from increased CSF flow based on the theory of Taylor dispersion (Taylor, 1953; Troyetsky et al., 2021), which describes the effective diffusion coefficient $D_{\text{eff}}$ characterizing the rate at which a tracer spreads in a shear flow because of the combined effect of advection and diffusion. For measured pial PVS size and flow speed (Mestre et al., 2018b) and a diffusion coefficient of $D=1\times 10^{-11}$ m^2^/s (Asgari et al., 2016), $D_{\text{eff}}/D=3.8$ (Figure S6A), suggesting pial CSF flow enhances transport 3.8-fold greater than diffusion alone. When the awake-to-sleep volume flow rate is increased less than 22% ($R_{\text{min}}$, $R_{\text{max}}$, and Intermediate scenario 2), the enhanced tracer transport is less than $D_{\text{eff}}^{\text{sleep}}/D_{\text{eff}}^{\text{awake}}=32$%, whereas a 3.2-fold increase in awake-to-sleep volume flow rate (Intermediate scenario 1 with ${\text{Γ}}_{\text{precap}}=0.07$) leads to $D_{\text{eff}}^{\text{sleep}}/D_{\text{eff}}^{\text{awake}}=300$% (Figure S6B). Note that prior studies computed enhancement factors based on oscillatory (zero mean) flow (Sharp et al., 2019; Asgari et al., 2016), whereas our calculations are based on steady (nonzero mean) flow, which we have previously argued is more effective for dispersive transport (Thomas, 2019; Troyetsky et al., 2021). Although Taylor dispersion in pial PVSs is unlikely to account for the entirety of tracer transport observed in experiments, these estimates generally suggest that Intermediate scenario 1 with small precapillary PVSs (${\text{Γ}}_{\text{precap}}=0.07$) is the only scenario with sleep/awake variations in volume flow rate large enough to explain tracer transport reported in several experiments (Xie et al., 2013; Plog et al., 2018; Hablitz et al., 2019; Stanton et al., 2021).

Overall, we find that parameters in the general range of Intermediate scenario 1 will satisfy the majority of experimental observations described in this article. We have found that a network with low PVS resistance (high PVS permeability) and high parenchymal resistance (whether from tight gaps between astrocyte endfeet, low parenchymal permeability, or both) requires a reasonably low pressure drop (Figure 2F), exhibits nearly perfect cortical CSF perfusion (Figures 3J and 3L), and – for small precapillary PVSs – most closely captures the observed increase in CSF influx during sleep compared to wakefulness (Figure 4M). In addition, Intermediate scenario 1 with ${\text{Γ}}_{\text{precap}}\approx 0.27$ is the only case which exhibits an equal 50/50 flow through precapillary PVSs and parenchyma. It is enticing to speculate that such a parameter regime may enable dynamic regulation of CSF transport; in this scenario, if parenchymal resistance were dominated by astrocyte endfeet, small changes in the endfoot gap could substantially shift CSF perfusion between slower parenchymal flow and faster precapillary PVSs flow. We caution that the parameter space is large, so Intermediate scenario 1 does not provide the only possible case that satisfies the aforementioned criteria, but rather points to a general parametric regime.

### Limitations of the study

There are numerous limitations in this study that are noteworthy. Perhaps the most consequential limitation is the uncertainty in several parameters that affect CSF transport through the glymphatic pathway, which we attempted to address by considering different limiting parametric scenarios. We restricted ourselves to a moderate number of cases for the sake of clarity, and we did so by lumping some parameters together, such as the astrocyte endfoot geometry and parenchymal permeability (Table 1). It is important to note that the parametric bounds we employ correspond to extreme values reported in or inferred from the literature (and therefore do not represent strict limits on feasible parameter ranges). Future experimental studies aimed at refining uncertain parameters will be of tremendous value for constructing predictive models. In particular, the hydraulic resistance of gaps between astrocyte endfeet is especially uncertain, with our estimates here ranging over almost seven orders of magnitude (Figure 3Y). The low end of this range suggests the astrocyte endfeet play no role in limiting CSF transport from the penetrating PVS to the parenchymal ECS (Figures 3S–3T and 3W–3X), whereas the upper limit has hydraulic resistance comparable to that of the parenchymal ECS (Figures 3Q–3R, 3U–3V), suggesting the astrocyte endfeet play a critical role. The bounds for endfeet gap sizes that we consider (20 nm–5.1 μm) come from studies based on chemical (Mathiisen et al., 2010) and cryo (Korogod et al., 2015) fixation. The former exhibits overlapping between the endfeet which is likely a consequence of shrinkage of the PVS during the fixation process (Mestre et al., 2018b), casting some doubt on this lower bound. However, a recent study (Wang et al., 2021) reported heterogeneity in the size of astrocyte endfeet, with larger endfeet (i.e., smaller and/or fewer gaps) surrounding larger vessels. This observation provides a mechanism that improves the uniformity of cortical CSF perfusion if the endfoot gaps are small enough to provide resistance comparable to that of the parenchymal ECS. Future studies should characterize astrocyte endfeet gap dimensions, ideally *in vivo*.

In our model, CSF flow is driven by the simplest possible mechanism – an externally applied pressure drop across the entire network. However, other potential driving mechanisms (e.g., pressure gradients generated by arterial pulsations (Mestre et al., 2018b), functional hyperemia (Kedarasetti et al., 2020), or osmotic effects (Plog et al., 2018; Halnes et al., 2019; Rasmussen et al., 2021)) could be tested with this network model approach by implementing pressure sources (i.e., “batteries”) throughout the network. In particular, incorporation of osmotic effects could be leveraged to investigate the mechanisms by which aquaporin-4 facilitates glymphatic flow (Iliff et al., 2012; Asgari et al., 2015; Mestre et al., 2018a; Hablitz et al., 2020), although there is some debate about this point (Smith et al., 2017; Mestre et al., 2018a). In this study, we have chosen an applied pressure drop in each scenario such that the median pial PVS flow speed matches the average value measured in experiments (Mestre et al., 2018b; Bedussi et al., 2017); future experiments that obtain flow speeds at multiple PVS locations will prove useful for validating the accuracy of our model. Yet another important limitation to our approach, already touched on in the fourth paragraph of the Discussion involves the connectivity of pial PVSs at the surface of the brain. By introducing “short-circuit” connections between PVSs of pial arteries and pial veins, our model could be adapted to estimate the fraction of CSF that continues along the surface of the brain (and/or through stomata) versus the fraction that continues through deeper PVSs and the parenchyma. Such a model would greatly benefit from experimental estimates of how many such connections typically exist. Finally, we highlight that our model can be generalized to predict transport of dye, metabolic waste, drugs, or any other molecules because of advection-diffusion. Such future studies will contribute to the substantial ongoing debate regarding the nature of transport in penetrating PVSs (Asgari et al., 2016; Sharp et al., 2019; Kedarasetti et al., 2020; Troyetsky et al., 2021).

In future work, we intend to implement numerous refinements to our simulation, but many will likely offer improvements that are of secondary importance compared to obtaining better estimates of critical parameters (as discussed above). The idealized geometry we have adopted has a regular, repeating structure composed of four different types of homogeneous channels and consequently lacks the high spatial variability characteristic of the true network. Future models could use randomly sampled statistical distributions to assign geometric parameters (Blinder et al., 2010, 2013) or directly implement the geometry of a synthetic (Linninger et al., 2019) or real (Kirst et al., 2020; Mestre et al., 2020) vascular network. We restricted our model to the arterial side of the network whereas we relied on assumptions about PVSs at the capillary and venous level to enable lumped modeling, but future studies could include substantially greater detail.

In this study, we predicted CSF transport throughout a mouse brain, but our network could be expanded to model a human brain by adding more vascular generations. Such an approach would be more challenging because of the fewer measurements available for constraining the parameter space in humans, compared to mice. However, many parameters may be conserved across species (e.g., porosity, PVS area ratios, endfoot gap size). Development of such a model has tremendous clinical value, as it could offer insight into a myriad of neurological disorders. Conditions such as Alzheimer’s disease, traumatic brain injury, and subarachnoid hemorrhage are all known to coincide with disrupted glymphatic transport (Rasmussen et al., 2018).

## STAR★Methods

### Key resources table

**Table:** 

REAGENT or RESOURCE	SOURCE	IDENTIFIER
**Software and algorithms**		

MATLAB	The MathWorks, Inc.	https://www.mathworks.com/products/matlab.html
Simulation codes	This work	https://doi.org/10.5281/zenodo.5644079

**Other**		

Pial artery segment length	Blinder et al. (2010)	https://doi.org/10.1073/pnas.1007239107
Pial artery diameter	Mestre et al. (2018b)	https://doi.org/10.1038/s41467-018-07318-3
Penetrating arteriole length (*l*_pen_)	Blinder et al. (2010)	https://doi.org/10.1073/pnas.1007239107
Penetrating arteriole diameter (*d*_pen_)	Blinder et al. (2010)	https://doi.org/10.1073/pnas.1007239107
Precapillary diameter (*d*_precap_)	Miyawaki et al., 2020	https://doi.org/10.1038/s41467-020-14786-z
Pial area ratio (Γ_pial_)	Mestre et al. (2018b)	https://doi.org/10.1038/s41467-018-07318-3
Precapillary area ratio (Γ_precap_)	Yurchenco (2011), Reitsma et al., (2007)	https://doi.org/10.1101/cshperspect.a004911, https://doi.org/10.1007/s00424-007-0212-8
Pial PVS permeability	Min Rivas et al. (2020)	https://doi.org/10.1098/rsif.2020.0593
Pen. & precap. permeability (*κ*_PVS_)	Basser (1992)	https://doi.org/10.1016/0026-2862(92)90077-3
Parenchymal permeability (*κ*_par_)	Holter et al. (2017), Basser (1992)	https://doi.org/10.1073/pnas.1706942114, https://doi.org/10.1016/0026-2862(92)90077-3
Median arteriole-to-venule distance (*l*_a−v_)	Blinder et al. (2013)	https://doi.org/10.1038/nn.3426
Pial PVS shape	Tithof et al. (2019)	https://doi.org/10.1186/s12987-019-0140-y
Penetrating PVS shape	Tithof et al. (2019)	https://doi.org/10.1186/s12987-019-0140-y
Precapillaries per arteriole (*n*)	Blinder et al. (2013)	https://doi.org/10.1038/nn.3426
Endfoot wall thickness (*T*)	Mathiisen et al. (2010)	https://doi.org/10.1002/glia.20990
Endfoot gap width (*g*)	Mathiisen et al. (2010), Korogod et al. (2015)	https://doi.org/10.1002/glia.20990, https://doi.org/10.7554/eLife.05793.001
Endfoot gap cavity fraction (*F*_*c*_)	Mathiisen et al. (2010), Korogod et al. (2015)	https://doi.org/10.1002/glia.20990, https://doi.org/10.7554/eLife.05793.001

### Resource availability

#### Lead contact

Further information and requests for resources should be directed to and will be fulfilled by the lead contact, Jeff Tithof (tithof@umn.edu).

#### Materials availability

This study did not generate new unique reagents.

### Method details

#### Numerical implementation

The network depicted in Figure 1A was inspired by a model proposed by Blinder et al. (2010). We used Matlab to develop the geometry, graphical representation, and computational modeling. First the spatial coordinates (for generating the schematic shown in Figure 1A), geometry, and connectivity of the network were generated and stored. This included vessel lengths, diameters, and types (pial, penetrating, precapillary, or parenchyma). The pressures and volume flow rates throughout the network were computed by enforcing Kirchhoff’s current law, ΣQ=0, at every node, where *Q* is the volumetric flow rate and summation is applied over all channels connected to a given node. To illustrate the implementation of this equation, consider three sequential nodes at pressures $p_{1}$, $p_{2}$, and $p_{3}$ connected by channels with conductance $c_{1,2}$ (which connects nodes 1 and 2) and $c_{2,3}$ (which connects nodes 2 and 3) . The volume flow rate from node 1 to node 2 is given by:

(Equation 2) $$Q_{1,2}=-c_{1,2}\left(p_{1}-p_{2}\right)$$

and the volume flow rate from node 2 to node 3 is given by:

(Equation 3) $$Q_{2,3}=c_{2,3}\left(p_{2}-p_{3}\right).$$

Kirchhoff’s current law requires that:

(Equation 4) $$Q_{1,2}+Q_{2,3}=-c_{1,2}\left(p_{1}-p_{2}\right)+c_{2,3}\left(p_{2}-p_{3}\right)=0,$$

which can be rewritten as

(Equation 5) $$Q_{1,2}+Q_{2,3}=-c_{1,2}p_{1}+\left(c_{1,2}+c_{2,3}\right)p_{2}-c_{2,3}p_{3}=0.$$

Enforcing Kirchhoff’s current law at every node in the network results in a linear algebra problem CP=z of the form:

$$\begin{array}{lll} \left[\begin{array}{llllll} c_{1,2} & -c_{1,2} & 0 & \cdots & & -1\\ -c_{1,2} & c_{1,2}+c_{2,3} & -c_{2,3} & \cdots & & 0\\ 0 & -c_{2,3} & \ddots & & & 0\\ \vdots & \vdots & & & & \vdots \\ & & & & & 0\\ & & & & & 1\\ 1 & 0 & \cdots & 0 & -1 & 0 \end{array}\right] & \left[\begin{array}{l} p_{1}\\ p_{2}\\ p_{3}\\ \vdots \\ p_{n-1}\\ p_{n}\\ Q_{\text{total}} \end{array}\right]= & \left[\begin{array}{l} 0\\ 0\\ 0\\ \vdots \\ 0\\ 0\\ \text{Δ}p_{\text{eff}} \end{array}\right] \end{array}$$

where $c_{i,j}$ are conductance values for the vessel connecting node *i* and *j*. Overall, the matrix *C* is sparse and was constructed by looping over each vessel segment connecting two nodes in the network and updating *C* with corresponding conductance values according to the connectivity of the network. Individual conductance values were computed as follows: for blood flow, Equation (1) was used along with a lumped model of the capillary and venous flow (see below); for CSF flow, we used power laws (Tithof et al., 2019) for non-porous pial and penetrating PVSs, the analytical solution for flow through a concentric circular annulus for non-porous precapillary PVSs (Equation (9) below), Darcy’s law for porous penetrating and precapillary PVSs, a lumped model for efflux routes (see below), and another lumped model for parenchymal flow (see below). The efflux node was grounded (as indicated in Figures 1B–1C) by setting the $n^{th}$ column of *C* to all zeroes. The vector *P* was obtained by computing the reduced row echelon form of [C|z].

Volumetric flow rates through each channel connected by nodes *i* and *j* were computed as $Q_{i,j}=c_{i,j}\left(p_{i}-p_{j}\right)$ and the corresponding average flow speed was computed as $Q_{i,j}/A_{i,j}$ where $A_{i,j}$ is the cross-sectional area of the given vessel or PVS (for parenchymal flow speeds, $A_{i,j}$ corresponds to the surface area of the outer wall of the penetrating PVS). To determine the external pressure drop $\text{Δ}p_{\text{eff}}$, for a given scenario, that results in a median pial CSF flow speed of 18.7 μm/s (Mestre et al., 2018b), we solved a root-finding problem. Since we want to determine the value of $\text{Δ}p_{\text{eff}}$ that satisfies the equation $v_{\text{model}}\left(\text{Δ}p_{\text{eff}}\right)=v_{\text{exp}}$, where $v_{\text{exp}}=18.7$μm/s and $v_{\text{model}}\left(\text{Δ}p_{\text{eff}}\right)$ is the median pial PVS flow speed obtained from the model, we subtract $v_{\text{model}}\left(\text{Δ}p_{\text{eff}}\right)$ from both sides and define the function $f\left(\text{Δ}p_{\text{eff}}\right)=v_{\text{exp}}-v_{\text{model}}\left(\text{Δ}p_{\text{eff}}\right)$, which we want to equal zero. We determined $\text{Δ}p_{\text{eff}}$ to an accuracy of four digits using the MATLAB function “fzero”; solving this root-finding problem typically required four iterations.

Flow fractions (Figure 2) were computed by first summing the total volumetric flow rate for either all parenchyma or all precapillary PVSs, then dividing by $Q_{\text{total}}$. Cumulative flow fractions at different cortical depths (Figure 3) were computed by summing a given volumetric flow rate (parenchyma, precapillary, or combined) for all locations at or above a given depth, then dividing by $Q_{\text{total}}$. Details are provided below describing how the change in parenchymal permeability was modeled for wakefulness relative to sleep. Total volumetric flow rates during wakefulness or sleep (Figures 4A–4H) were computed in a given scenario by summing the volumetric flow rates over the entire network for a given route (parenchyma, precapillary PVSs, or both), and each corresponding sleep/wake ratio (Figures 4I–4P) was then computed.

In addition to the characterization of the network geometry idealization provided by the blood flow simulations (Figures 1D–1F), we also verified our numerical methods by testing the rotational symmetry of the network, which suggests that we are indeed implementing and solving the geometry that we intend to. By implementing a total of three inlets (which is non-physiological), the network exhibits a $120^{\circ }$ rotational symmetry (Figure S7). We computed the relative error for each node by computing the relative error in pressure $\left|p_{i}-{p^{\text{′}}}_{i}\right|/p_{i}$, where *i* is the node index and the prime indicates the $120^{\circ }$-rotated network. This calculation showed that the largest deviation from rotational symmetry is $4.3\times 10^{-9}$%. We also verified the volumetric flow rate through each pial offshoot (i.e., the pial bifurcation leading to a penetrating PVS) by comparing $\text{Δ}p_{\text{offshoot}}/Q_{\text{offshoot}}$ to the equivalent lumped resistance for each offshoot, computed analytically. Here, $\text{Δ}p_{\text{offshoot}}$ is the pressure drop between the start of each offshoot and ground and $Q_{\text{offshoot}}$ is the total volumetric flow rate through a given offshoot. We find agreement in all cases to within $5.6\times 10^{-5}$%.

#### Lumped model of capillary bed and venous resistance for blood flow

Blinder et al. (2013) found that the resistance across nodes in the three-dimensional resistive network of the capillary bed asymptotes to a constant value with increasing distance between nodes. They found that the asymptotic resistance is numerically the same as a network with a resistance value of $2\times 10^{7}$ mmHg⋅min/ml and that the average resistance for penetrating venules from the surface to the cortical depth layer of 4 was $2.5\times 10^{6}$ mmHg⋅min/ml. Accordingly, we used a value of $2.3\times 10^{7}$ mmHg⋅min/ml to represent the resistance to flow through the capillary bed and venous circulation back to the heart (to ground, in the circuit analogy), indicated by the gray$R_{\text{efflux}}$ resistors in Figure 1C. In this diagram, the green resistor represents the resistance to flow through a single precapillary segment, and the green symbols in Figure 1D indicate the pressure at the distal end of that single precapillary segment.

#### Lumped model of capillary and venous PVS resistance for perivascular CSF flow

We modeled the resistance through the capillary PVSs based on the idea that the entire vascular capillary bed could be represented by a single equivalent resistor, as described by Blinder et al. (2013). We first computed the effective precapillary length using Equation (1), with $5\times 10^{7}$ mmHg⋅min/ml and r=2μm, consistent with the values used by Blinder et al. We used the value we obtained (202 μm) to calculate the equivalent perivascular resistance. This equivalent resistance, $R_{\text{precap}}$, represents the resistance to flow through the entire network of capillary PVSs beyond each given precapillary and is represented by each green resistor shown in Figure 1C. The resistance to flow through the venous PVS, $R_{\text{efflux}}$, is assumed to be negligible and is arbitrarily set as 1 mmHg⋅min/ml ($R_{\text{efflux}}$ is represented by the gray resistors in Figure 1C). It should be noted that this approach differs from the idealized vascular model, where $R_{\text{precap}}$ represents flow through a single precapillary and $R_{\text{efflux}}$ represents flow through the remainder of the capillary bed and the venous circulation.

#### Lumped model of parenchymal flow

The parenchyma was modeled as a porous medium with two-dimensional planar flow from penetrating arterioles to ascending veins. The total resistance to flow, $R_{\text{par}}$, was modeled as two resistors in series, representing the resistance to flow through the gaps in the astrocyte endfeet surrounding the penetrating arteriole, $R_{\text{AE}}$, and the resistance to flow through the surrounding extracellular space, $R_{\text{ECS}}$, so that $R_{\text{par}}=R_{\text{AE}}+R_{\text{ECS}}$. Estimates for the cavity fraction, endfeet gap width, and parenchymal permeability, which are used to calculate $R_{\text{AE}}$ and $R_{\text{ECS}}$ as described below, differ widely depending on the approach used to estimate them. Therefore, in order to bracket a reasonable range of expected flows, a high resistance (small cavity fraction/endfeet gap and small permeability) and a low resistance (large cavity fraction/endfeet gap and high permeability) case are modeled based on a range of estimates from the literature.

The resistance to flow through the gaps in the endfeet was modeled as flow between infinite parallel plates, for which$R=12\mu T/g^{3}l$, where *T* and *g* are the thickness (dimension parallel to flow) of the gap and gap width, respectively, as shown in Figure S2. The length of the gap, *l*, was estimated by setting the area of the gap equal to the product of the cavity fraction of the gap and area of the penetrating arteriole segment through which CSF would flow, or $lg=F_{c}\pi \left(d_{pen}\sqrt{{\text{Γ}}_{\text{pen}}+1}\right)l_{pen}/n$, where $F_{c}$, $d_{pen}$, ${\text{Γ}}_{\text{pen}}$, $l_{pen}$, and *n* are the cavity fraction of the endfeet gaps, diameter of the penetrating arterioles, PVS-to-arteriole area ratio, length of the penetrating arterioles, and number of precapillaries per arteriole, respectively. Note that $d_{pen}\sqrt{{\text{Γ}}_{\text{pen}}+1}$ is equivalent to the diameter of the outer wall of the PVS. The resistance to flow through the endfeet gaps is then calculated as

(Equation 6) $$R_{AE}=\frac{12\mu T}{g^{2}F_{c}\pi \left(d_{pen}\sqrt{{\text{Γ}}_{\text{pen}}+1}\right)\left(l_{pen}/n\right)}.$$

For the high resistance case, the endfoot gap and cavity fraction are assumed to be 20 nm and 0.3% based on electron microscopy measurements obtained by Mathiisen et al. (2010). Their measurements were obtained using tissue that was chemically fixed, which has been shown to significantly alter these dimensions (Korogod et al., 2015). Nevertheless, their measurements have been used in other studies modeling the resistance to flow into the parenchyma and are included as an upper bound on the expected resistance. Korogod et al. (2015) compared cryogenic and chemical fixation, and found significant differences in endfeet cavity fraction (37% vs 4%). For the low resistance case, we used the endfoot gap cavity fraction estimated from cryogenic fixation, 37%. Mathiisen et al. (2010) estimated the cavity fraction they reported as $F_{c}=gN/\pi d_{pen}$, where *N* is the average number of transected endfoot gaps per vessel profile, which they reported as 2.5. Since the density of endfeet gaps is unlikely to change with chemical fixation, we assumed the same relationship and used N=2.5 to estimate an endfoot gap width of 5.1 μm for the low resistance case. In this case, $R_{AE}$ was so small relative to $R_{ECS}$ that it could be considered negligible (Figures 3S, 3T, 3W, and 3X), meaning that the endfeet resist flow far less than the parenchyma.

The resistance to flow through the extracellular space, modeled as flow between a point source with constant flux to a sink, was calculated as described by Holter et al. in their Supporting Information (Holter et al., 2017):

(Equation 7) $$R_{\text{ECS}}=\frac{\mu \text{ln}\left({\left[1-2\left(l_{a-v}/d_{pen}\right)\right]}^{2}\right)}{2\pi \kappa_{par}\left(l_{pen}/n\right)},$$

where $R_{\text{ECS}}$ is the parenchymal resistance and $l_{a-v}$ is the median distance between an arteriole and the nearest venule. The quantity $l_{pen}/n$ indicates the length of the penetrating arteriole segment since the expression provided by Holter et al. was for a flux per unit length.

Equivalent permeability for flow through an open (non-porous) annulus

We modeled flow through the penetrating and precapillary PVSs using Darcy’s law:

(Equation 8) $$Q=-\frac{\kappa A_{\text{PVS}}}{\mu }\nabla p,$$

where *Q* is the volume flow rate, κ is the permeability, $A_{\text{PVS}}$ is the PVS cross-sectional area, μ is the dynamic viscosity, and *p* is the pressure. To calculate the upper bound in permeability, we considered the volume flow rate through a (non-porous) concentric circular annulus, given by Equations 3–51 in White (2006):

(Equation 9) $$Q=\frac{\pi }{8\mu }\left(-\frac{dp}{dz}\right)\left[r_{2}^{4}-r_{1}^{4}-\frac{{\left(r_{2}^{2}-r_{1}^{2}\right)}^{2}}{\text{ln}\left(r_{2}/r_{1}\right)}\right],$$

where $r_{2}$ is the radius of the outer circle (outer PVS wall) and $r_{1}$ is the radius of the inner circle (blood vessel). Noting that $A_{PVS}=\pi \left(r_{2}^{2}-r_{1}^{2}\right)$, setting Equations (8) and (9) equal, and then solving for κ, one obtains:

(Equation 10) $$\kappa =\frac{1}{8}\left[r_{2}^{2}+r_{1}^{2}-\frac{r_{2}^{2}-r_{1}^{2}}{\text{ln}\left(r_{2}/r_{1}\right)}\right].$$

Hence, Equation (10) provides the upper bound for $\kappa_{PVS}$ used throughout this article which is equivalent to modeling an open (non-porous) PVS.

Change in parenchymal permeability for wake versus sleep

The Kozeny-Carman equation is:

(Equation 11) $$\kappa =\frac{\varepsilon^{3}}{\tau {\left(1-\varepsilon \right)}^{2}S^{2}},$$

where ε is the porosity, τ is the tortuosity, and *S* is the specific surface area for a porous medium (Hommel et al., 2018). Xie et al. (2013) reported an increase of ε from 0.14 during wakefulness to 0.23 during sleep, with no change in tortuosity. Assuming *S* remains approximately constant, this suggests $\kappa_{\text{par}}^{\text{sleep}}/\kappa_{\text{par}}^{\text{wake}}=5.5$.

## Data Availability

• Simulation codes are available at https://doi.org/10.5281/zenodo.5644079. • All the data reported in this paper will be shared by the lead contact upon request. • Any additional information required to reanalyze the data reported in this paper is available from the lead contact upon request.

## References

[bib1] Adams M.D., Winder A.T., Blinder P., Drew P.J. (2018). The pial vasculature of the mouse develops according to a sensory-independent program. Sci. Rep..

[bib2] Asgari M., De Zélicourt D., Kurtcuoglu V. (2015). How astrocyte networks may contribute to cerebral metabolite clearance. Sci. Rep..

[bib3] Asgari M., de Zélicourt D., Kurtcuoglu V. (2016). Glymphatic solute transport does not require bulk flow. Sci. Rep..

[bib4] Basser P.J. (1992). Interstitial pressure, volume, and flow during infusion into brain tissue. Microvasc. Res..

[bib5] Bedussi B., Almasian M., de Vos J., VanBavel E., Bakker E.N.T.P. (2017). Paravascular spaces at the brain surface: low resistance pathways for cerebrospinal fluid flow. J. Cerebr. Blood F. Met..

[bib6] Benveniste H., Liu X., Koundal S., Sanggaard S., Lee H., Wardlaw J. (2019). The glymphatic system and waste clearance with brain aging: a review. Gerontology.

[bib7] Blinder P., Shih A.Y., Rafie C., Kleinfeld D. (2010). Topological basis for the robust distribution of blood to rodent neocortex. Proc. Nat. Acad. Sci. U S A.

[bib8] Blinder P., Tsai P.S., Kaufhold J.P., Knutsen P.M., Suhl H., Kleinfeld D. (2013). The cortical angiome: an interconnected vascular network with noncolumnar patterns of blood flow. Nat. Neurosci..

[bib9] Bobo R.H., Laske D.W., Akbasak A., Morrison P.F., Dedrick R.L., Oldfield E.H. (1994). Convection-enhanced delivery of macromolecules in the brain. Proc. Natl. Acad. Sci. U S A.

[bib10] Cserr H.F., Cooper D.N., Suri P.K., Patlak C.S. (1981). Efflux of radiolabeled polyethylene glycols and albumin from rat brain. Am. J. Physiol.–Renal.

[bib11] Du T., Mestre H., Kress B.T., Liu G., Sweeney A.M., Samson A.J., Rasmussen M.K., Mortensen K.N., Bork P.A.R., Peng W. (2021). Cerebrospinal fluid is a significant fluid source for anoxic cerebral oedema. Brain.

[bib12] Eide P.K., Vinje V., Pripp A.H., Mardal K.A., Ringstad G. (2021). Sleep deprivation impairs molecular clearance from the human brain. Brain.

[bib13] Faghih M.M., Sharp M.K. (2018). Is bulk flow plausible in perivascular, paravascular and paravenous channels?. Fluids Barriers CNS.

[bib14] Fultz N.E., Bonmassar G., Setsompop K., Stickgold R.A., Rosen B.R., Polimeni J.R., Lewis L.D. (2019). Coupled electrophysiological, hemodynamic, and cerebrospinal fluid oscillations in human sleep. Science.

[bib15] Gaberel T., Gakuba C., Goulay R., De Lizarrondo S.M., Hanouz J.L., Emery E., Touze E., Vivien D., Gauberti M. (2014). Impaired glymphatic perfusion after strokes revealed by contrast-enhanced mri: a new target for fibrinolysis?. Stroke.

[bib16] Gu X., Song Q., Zhang Q., Huang M., Zheng M., Chen J., Wei D., Chen J., Wei X., Chen H., Zheng G., Gao X. (2020). Clearance of two organic nanoparticles from the brain via the paravascular pathway. J. Control Release.

[bib17] Hablitz L.M., Plá V., Giannetto M., Vinitsky H.S., Stæger F.F., Metcalfe T., Nguyen R., Benrais A., Nedergaard M. (2020). Circadian control of brain glymphatic and lymphatic fluid flow. Nat. Commun..

[bib18] Hablitz L.M., Vinitsky H.S., Sun Q., Stæger F.F., Sigurdsson B., Mortensen K.N., Lilius T.O., Nedergaard M. (2019). Increased glymphatic influx is correlated with high EEG delta power and low heart rate in mice under anesthesia. Sci. Adv.

[bib19] Halnes G., Pettersen K.H., Øyehaug L., Rognes M.E., Einevoll G.T. (2019). Computational Glioscience.

[bib20] Hannocks M.J., Pizzo M.E., Huppert J., Deshpande T., Abbott N.J., Thorne R.G., Sorokin L. (2018). Molecular characterization of perivascular drainage pathways in the murine brain. J. Cerebr. Blood F. Met..

[bib21] Hladky S.B., Barrand M.A. (2018). Elimination of substances from the brain parenchyma: efflux via perivascular pathways and via the blood–brain barrier. Fluids Barriers CNS.

[bib22] Holter K.E., Kehlet B., Devor A., Sejnowski T.J., Dale A.M., Omholt S.W., Ottersen O.P., Nagelhus E.A., Mardal K.A., Pettersen K.H. (2017). Interstitial solute transport in 3D reconstructed neuropil occurs by diffusion rather than bulk flow. Proc. Nat. Acad. Sci. U S A.

[bib23] Hommel J., Coltman E., Class H. (2018). Porosity–permeability relations for evolving pore space: a review with a focus on (bio-)geochemically altered porous media. Transp. Porous Med..

[bib24] Iliff J.J., Wang M., Zeppenfeld D.M., Venkataraman A., Plog B.A., Liao Y., Deane R., Nedergaard M. (2013). Cerebral arterial pulsation drives paravascular CSF-interstitial fluid exchange in the murine brain. J. Neurosci..

[bib25] Iliff J.J., Wang M., Liao Y., Plogg B.A., Peng W., Gundersen G.A., Benveniste H., Vates G.E., Deane R., Goldman S.A. (2012). A paravascular pathway facilitates CSF flow through the brain parenchyma and the clearance of interstitial solutes, including amyloid β. Sci. Transl. Med..

[bib26] Jin B.J., Smith A.J., Verkman A.S. (2016). Spatial model of convective solute transport in brain extracellular space does not support a “glymphatic” mechanism. Gen. Physiol..

[bib27] Karimy J.K., Kahle K.T., Kurland D.B., Yu E., Gerzanich V., Simard J.M. (2015). A novel method to study cerebrospinal fluid dynamics in rats. J. Neurosci. Meth..

[bib28] Kedarasetti R.T., Turner K.L., Echagarruga C., Gluckman B.J., Drew P.J., Costanzo F. (2020). Functional hyperemia drives fluid exchange in the paravascular space. Fluids Barriers CNS.

[bib29] Kirst C., Skriabine S., Vieites-Prado A., Topilko T., Bertin P., Gerschenfeld G., Verny F., Topilko P., Michalski N., Tessier-Lavigne M., Renier N. (2020). Mapping the fine-scale organization and plasticity of the brain vasculature. Cell.

[bib30] Korogod N., Petersen C.C.H., Knott G.W. (2015). Ultrastructural analysis of adult mouse neocortex comparing aldehyde perfusion with cryo fixation. eLife.

[bib31] Koundal S., Elkin R., Nadeem S., Xue Y., Constantinou S., Sanggaard S., Liu X., Monte B., Xu F., Van Nostrand W. (2020). Optimal mass transport with Lagrangian workflow reveals advective and diffusion driven solute transport in the glymphatic system. Sci. Rep..

[bib32] Lee H., Mortensen K., Sanggaard S., Koch P., Brunner H., Quistorff B., Nedergaard M., Benveniste H. (2018). Quantitative Gd-DOTA uptake from cerebrospinal fluid into rat brain using 3D VFA-SPGR at 9.4 T. Magn. Reson. Med..

[bib33] Linninger A., Hartung G., Badr S., Morley R. (2019). Mathematical synthesis of the cortical circulation for the whole mouse brain-part I. theory and image integration. Comput. Biol. Med..

[bib34] Liu G., Mestre H., Sweeney A.M., Sun Q., Weikop P., Du T., Nedergaard M. (2020). Direct measurement of cerebrospinal fluid production in mice. Cell Rep.

[bib35] Ma Q., Ries M., Decker Y., Müller A., Riner C., Bücker A., Fassbender K., Detmar M., Proulx S.T. (2019). Rapid lymphatic efflux limits cerebrospinal fluid flow to the brain. Acta Neuropathol..

[bib36] Martinac A.D., Bilston L.E. (2020). Computational modelling of fluid and solute transport in the brain. Biomech. Model. Mechan..

[bib37] Mathiisen T.M., Lehre K.P., Danbolt N.C., Ottersen O.P. (2010). The perivascular astroglial sheath provides a complete covering of the brain microvessels: an electron microscopic 3D reconstruction. Glia.

[bib38] Mattson D.L. (2001). Comparison of arterial blood pressure in different strains of mice. Am. J. Hypertens..

[bib39] Mestre H., Du T., Sweeney A.M., Liu G., Samson A.J., Peng W., Mortensen K.N., Stæger F.F., Bork P.A.R., Bashford L. (2020). Cerebrospinal fluid influx drives acute ischemic tissue swelling. Science.

[bib40] Mestre H., Hablitz L.M., Xavier A.L.R., Feng W., Zou W., Pu T., Monai H., Murlidharan G., Pike M.M., Pla V. (2018). Aquaporin-4-dependent glymphatic solute transport in the rodent brain. eLife.

[bib41] Mestre H., Tithof J., Du T., Song W., Peng W., Sweeney A.M., Olveda G., Thomas J.H., Nedergaard M., Kelley D.H. (2018). Flow of cerebrospinal fluid is driven by arterial pulsations and is reduced in hypertension. Nat. Commun..

[bib42] Milhorat T.H. (1975). The third circulation revisited. J. Neurosurg..

[bib43] Min Rivas F., Liu J., Martell B.C., Du T., Mestre H., Nedergaard M., Tithof J., Thomas J.H., Kelley D.H. (2020). Surface periarterial spaces of the mouse brain are open, not porous. J. R. Soc. Interf..

[bib79] Miyawaki T., Morikawa S., Susaki E.A. (2020). Visualization and molecular characterization of whole-brain vascular networks with capillary resolution. Nat Commun.

[bib44] Morrison P.F., Laske D.W., Bobo H., Oldfield E.H., Dedrick R.L. (1994). High-flow microinfusion: tissue penetration and pharmacodynamics. Am. J. Physiol. – Reg..

[bib45] Nedergaard M., Goldman S.A. (2020). Glymphatic failure as a final common pathway to dementia. Science.

[bib46] Neeves K.B., Lo C.T., Foley C.P., Saltzman W.M., Olbricht W.L. (2006). Fabrication and characterization of microfluidic probes for convection enhanced drug delivery. J. Control Release.

[bib47] Penn R.D., Linninger A. (2009). The physics of hydrocephalus. Pediatr. Neurosurg..

[bib48] Pizzo M.E., Wolak D.J., Kumar N.N., Brunette E., Brunnquell C.L., Hannocks M.J., Abbott N.J., Meyerand M.E., Sorokin L., Stanimirovic D.B., Thorne R.G. (2018). Intrathecal antibody distribution in the rat brain: surface diffusion, perivascular transport and osmotic enhancement of delivery. J. Physiol..

[bib49] Plog B.A., Mestre H., Olveda G.E., Sweeney A.M., Kenney H.M., Cove A., Dholakia K.Y., Tithof J., Nevins T.D., Lundgaard I., Du T., Kelley D.H., Nedergaard M. (2018). Transcranial optical imaging reveals a pathway for optimizing the delivery of immunotherapeutics to the brain. JCI insight.

[bib50] Plog B.A., Nedergaard M. (2018). The glymphatic system in central nervous system health and disease: past, present, and future. Annu. Rev. Pathol.-Mech..

[bib51] Prabhu S.S., Broaddus W.C., Gillies G.T., Loudon W.G., Chen Z.J., Smith B. (1998). Distribution of macromolecular dyes in brain using positive pressure infusion: a model for direct controlled delivery of therapeutic agents. Surg. Neurol..

[bib52] Raghunandan A., Ladron-de Guevara A., Tithof J., Mestre H., Du T., Nedergaard M., Thomas J.H., Kelley D.H. (2021). Bulk flow of cerebrospinal fluid observed in periarterial spaces is not an artifact of injection. eLife.

[bib53] Rasmussen M.K., Mestre H., Nedergaard M. (2021). Fluid transport in the brain. Physiol. Rev..

[bib54] Rasmussen M.K., Mestre H., Nedergaard M. (2018). The glymphatic pathway in neurological disorders. Lancet Neurol..

[bib55] Ray L., Iliff J.J., Heys J.J. (2019). Analysis of convective and diffusive transport in the brain interstitium. Fluids Barriers CNS.

[bib56] Ray L.A., Heys J.J. (2019). Fluid flow and mass transport in brain tissue. Fluids.

[bib57] Reitsma S., Slaaf D.W., Vink H., Van Zandvoort M.A.M.J., oude Egbrink M.G.A. (2007). The endothelial glycocalyx: composition, functions, and visualization. Pflüg. Arch. Eur. J. Phy..

[bib58] Rennels M.L., Gregory T.F., Blaumanis O.R., Fujimoto K., Grady P.A. (1985). Evidence for a ‘paravascular’ fluid circulation in the mammalian central nervous system, provided by the rapid distribution of tracer protein throughout the brain from the subarachnoid space. Brain Res..

[bib59] Rey J., Sarntinoranont M. (2018). Pulsatile flow drivers in brain parenchyma and perivascular spaces: a resistance network model study. Fluids Barriers CNS.

[bib60] Ringstad G., Valnes L.M., Dale A.M., Pripp A.H., Vatnehol S.A.S., Emblem K.E., Mardal K.A., Eide P.K. (2018). Brain-wide glymphatic enhancement and clearance in humans assessed with MRI. JCI Insight.

[bib61] Roberts K.F., Elbert D.L., Kasten T.P., Patterson B.W., Sigurdson W.C., Connors R.E., Ovod V., Munsell L.Y., Mawuenyega K.G., Miller-Thomas M.M. (2014). Amyloid-β efflux from the central nervous system into the plasma. Ann. Neurol..

[bib62] Schain A.J., Melo-Carrillo A., Strassman A.M., Burstein R. (2017). Cortical spreading depression closes the paravascular space and impairs glymphatic flow: implications for migraine headache. J. Neurosci..

[bib63] Keith Sharp M., Carare R.O., Martin B.A. (2019). Dispersion in porous media in oscillatory flow between flat plates: applications to intrathecal, periarterial and paraarterial solute transport in the central nervous system. Fluids Barriers CNS.

[bib64] Shokri-Kojori E., Wang G.J., Wiers C.E., Demiral S.B., Guo M., Kim S.W., Lindgren E., Ramirez V., Zehra A., Freeman C., Miller G., Manza P., Srivastava T., De Santi S., Tomasi D., Benveniste H., Volkow N.D. (2018). β-amyloid accumulation in the human brain after one night of sleep deprivation. Proc. Natl. Acad. Sci. U S A.

[bib65] Smith A.J., Yao X., Dix J.A., Jin B.J., Verkman A.S. (2017). Test of the ‘glymphatic’ hypothesis demonstrates diffusive and aquaporin-4-independent solute transport in rodent brain parenchyma. eLife.

[bib66] Smith J.H., Humphrey J.A.C. (2007). Interstitial transport and transvascular fluid exchange during infusion into brain and tumor tissue. Microvasc. Res..

[bib67] Stanton E.H., Persson N.D.Å., Gomolka R.S., Lilius T., Sigurðsson B., Lee H., Xavier A.L.R., Benveniste H., Nedergaard M., Mori Y. (2021). Mapping of CSF transport using high spatiotemporal resolution dynamic contrast-enhanced MRI in mice: effect of anesthesia. Magn. Reson. Med..

[bib68] Sullan M.J., Asken B.M., Jaffee M.S., DeKosky S.T., Bauer R.M. (2018). Glymphatic system disruption as a mediator of brain trauma and chronic traumatic encephalopathy. Neurosci. Biobehav. R..

[bib69] Taoka T., Naganawa S. (2020). Glymphatic imaging using MRI. J. Magn. Reson. Imaging.

[bib70] Taylor G.I. (1953). Dispersion of soluble matter in solvent flowing slowly through a tube. P. R. Soc. A..

[bib71] Thomas J.H. (2019). Fluid dynamics of cerebrospinal fluid flow in perivascular spaces. J. R. Soc. Interf..

[bib72] Tithof J., Kelley D.H., Mestre H., Nedergaard M., Thomas J.H. (2019). Hydraulic resistance of periarterial spaces in the brain. Fluids Barriers CNS.

[bib73] Troyetsky D.E., Tithof J., Thomas J.H., Kelley D.H. (2021). Dispersion as a waste-clearance mechanism in flow through penetrating perivascular spaces in the brain. Sci. Rep..

[bib74] Vinje V., Eklund A., Mardal K.A., Rognes M.E., Støverud K.H. (2020). Intracranial pressure elevation alters CSF clearance pathways. Fluids Barriers CNS.

[bib75] Wang M.X., Ray L., Tanaka K.F., Iliff J.J., Heys J. (2021). Varying perivascular astroglial endfoot dimensions along the vascular tree maintain perivascular-interstitial flux through the cortical mantle. Glia.

[bib76] White F.M. (2006).

[bib77] Xie L., Kang H., Xu Q., Chen M.J., Liao Y., Thiyagarajan M., O’Donnell J., Christensen D.J., Nicholson C., Iliff J.J. (2013). Sleep drives metabolite clearance from the adult brain. Science.

[bib78] Yurchenco P.D. (2011). Basement membranes: cell scaffoldings and signaling platforms. CSH Perspect. Biol..

