# Supplementary material for: A network model of glymphatic flow under different experimentally-motivated parametric scenarios
Source: iScience. 2022 Apr 14;25(5):104258. doi: 10.1016/j.isci.2022.104258 (PMC9062681; doi:10.1016/j.isci.2022.104258)
Supplement: Supplementary file 1 — Document S1. Figures S1–S7 and Table 1 [file mmc1.pdf]

iScience, Volume *25*

Supplemental information

A network model of glymphatic flow under different experimentally-motivated parametric scenarios

Jeffrey Tithof, Kimberly A.S. Boster, Peter A.R. Bork, Maiken Nedergaard, John H. Thomas, and Douglas H. Kelley

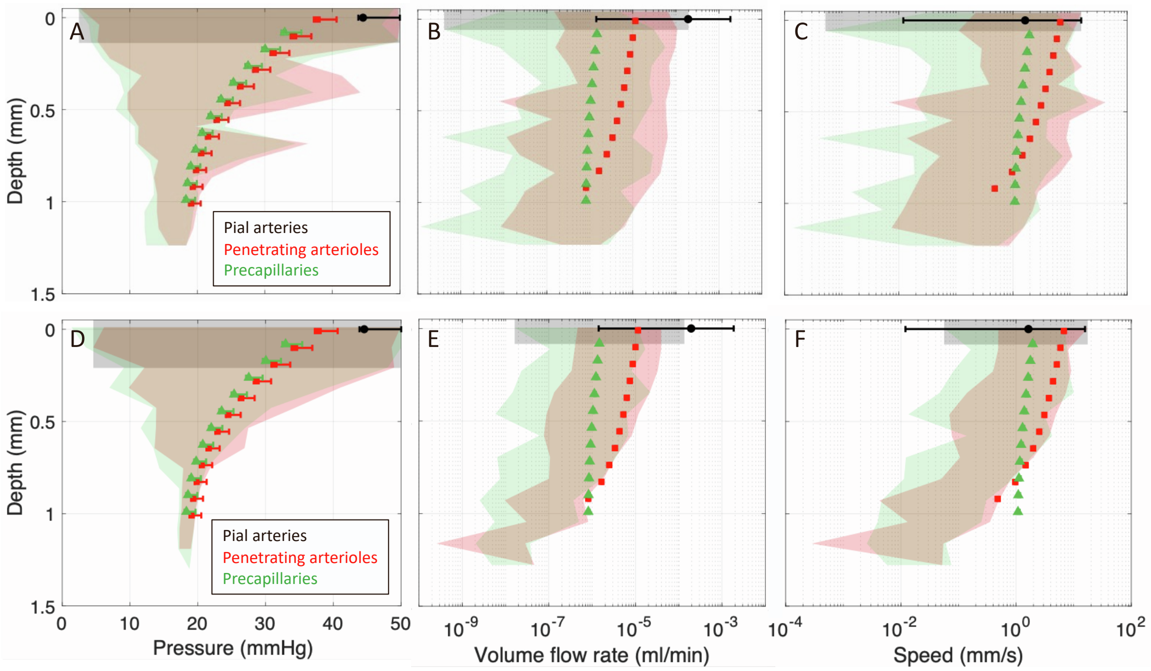

Figure S1: Comparisons of blood flow in the idealized model for two additional mice, Related to Figure 1. Plots of (A, D) pressure, (B, E) volume flow rate, and (C, F) speed for blood flow in two more mice. The shaded regions indicate the range of values for a real vascular topology reported by Blinder et al (2013), while the symbols and error bars indicate the mean and range of values, respectively, computed using the idealized geometry.

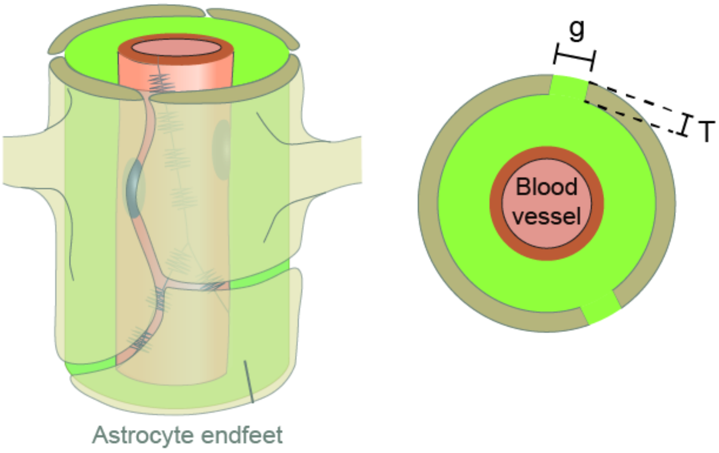

Figure S2: Idealized geometry of the gaps between endfeet, Related to Table 1. In our model, CSF leaves the perivascular space (green) surrounding penetrating arterioles (red) via gaps of width g and thickness T. The gaps between endfeet are long and narrow, as described by Wang et al (2021). To estimate the hydraulic resistance of the gaps, we consider flow between infinite parallel plates.

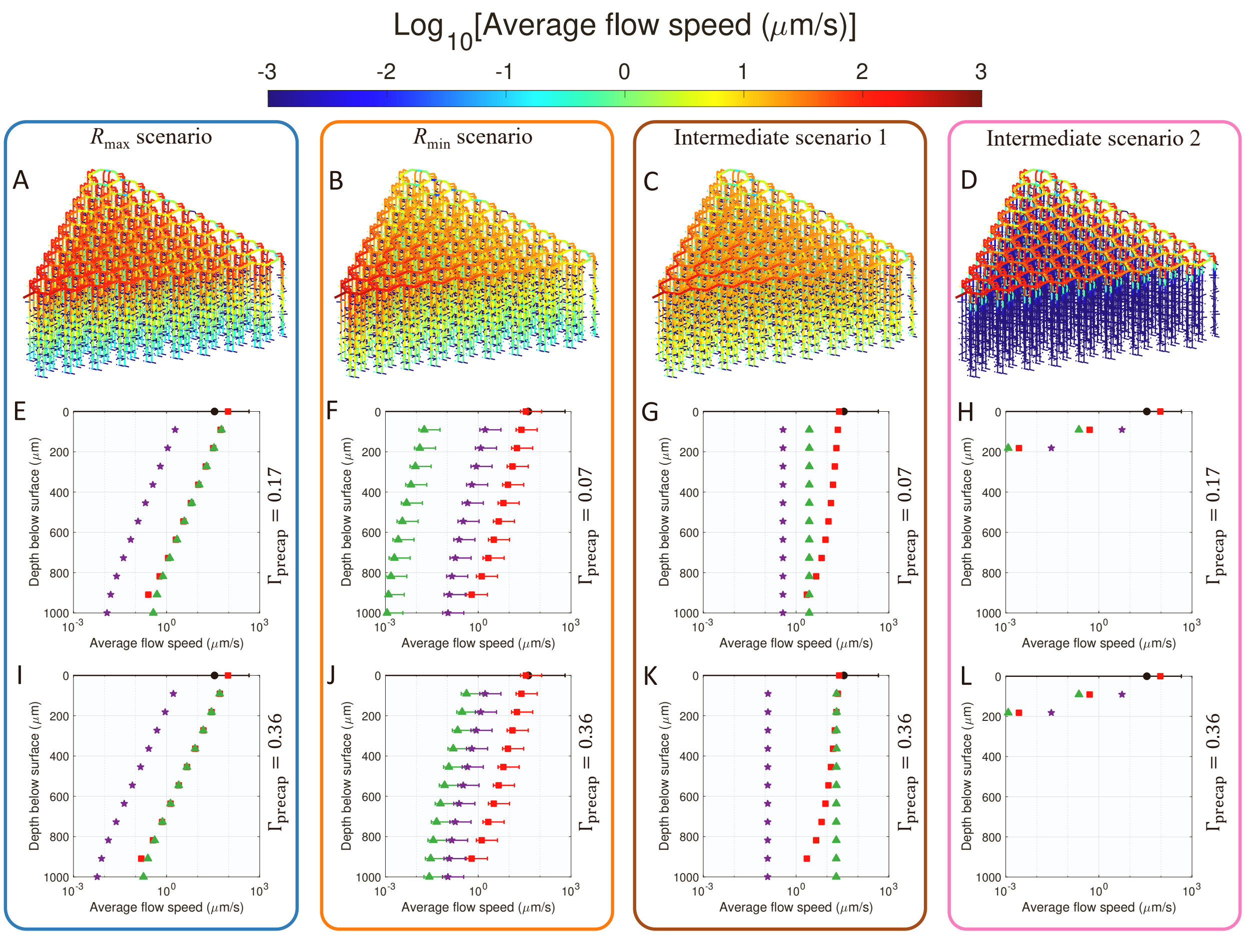

Figure S3: Spatial variation of flow speed, Related to Figure 3. (A-D) Schematic diagrams of the network model with color indicating the mean flow speed. The scenarios are indicated at the top of each box; only the case of small precapillary PVSs is plotted ((A,D) $\Gamma_{\text{precap}} = 0.17$ and (B, C) $\Gamma_{\text{precap}} = 0.07$) which is virtually indistinguishable from the large precapillary PVS case. (E-L) Plots of average flow speed across the depth of the cortex. The error bars indicate the range of the data, and the area ratio of precapillary PVSs Γ_{precap} is indicated to the right of each plot.

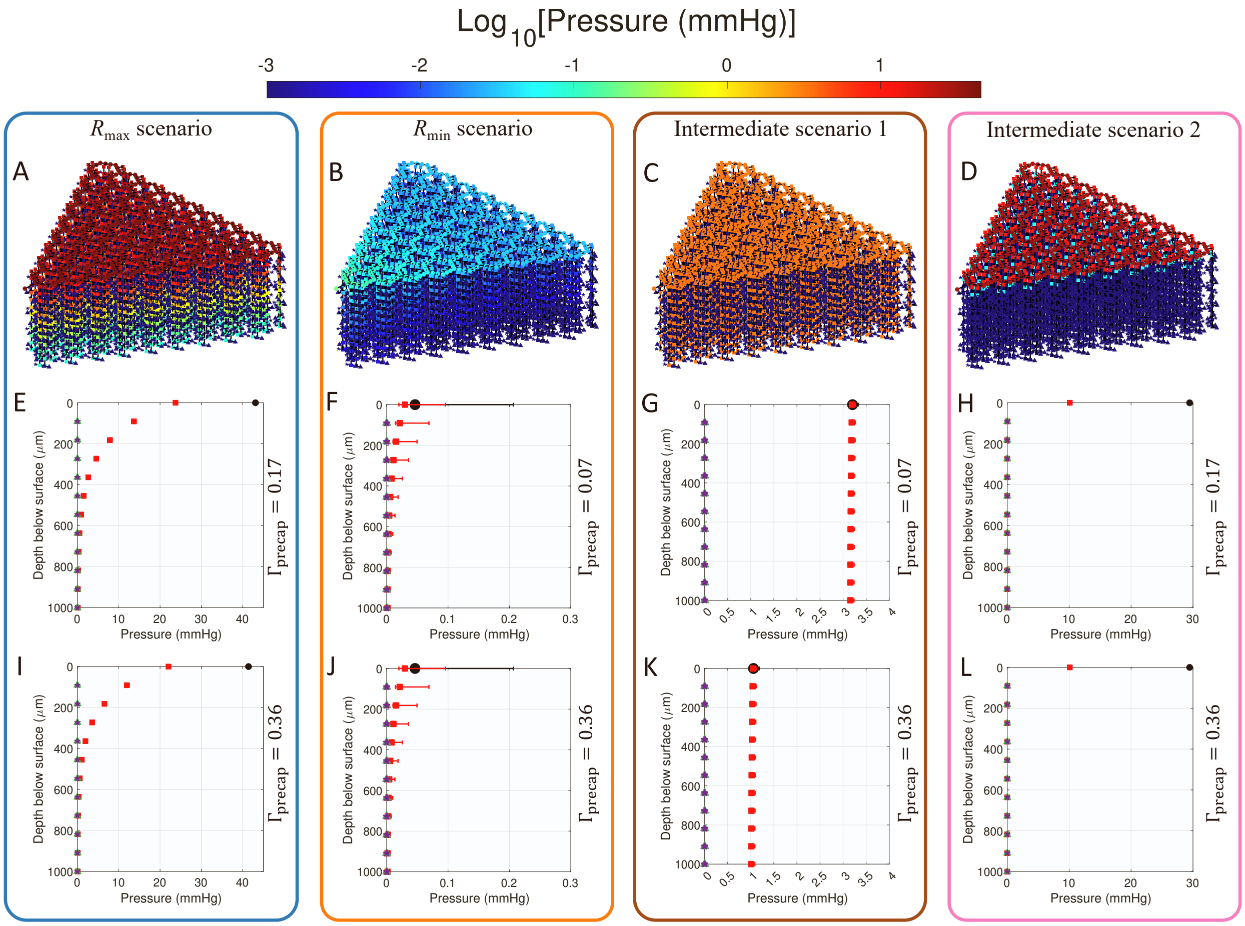

Figure S4: Spatial variation of pressure, Related to Figure 3. (A-D) Schematic diagrams of the network model with color indicating the pressure, plotted on a log scale (indicated by the color bar at the top). The scenarios are indicated at the top of each box; only the case of small precapillary PVSs is plotted ((A,D) $\Gamma_{\text{precap}} = 0.17$ and (B, C) $\Gamma_{\text{precap}} = 0.07$) which is virtually indistinguishable from the large precapillary PVS case. (E-L) Plots of pressure across the depth of the cortex, on a linear scale. The error bars indicate the range of the data, and the area ratio of precapillary PVSs Γ_{precap} is indicated to the right of each plot. Note the x-axis limits vary for each different scenario.

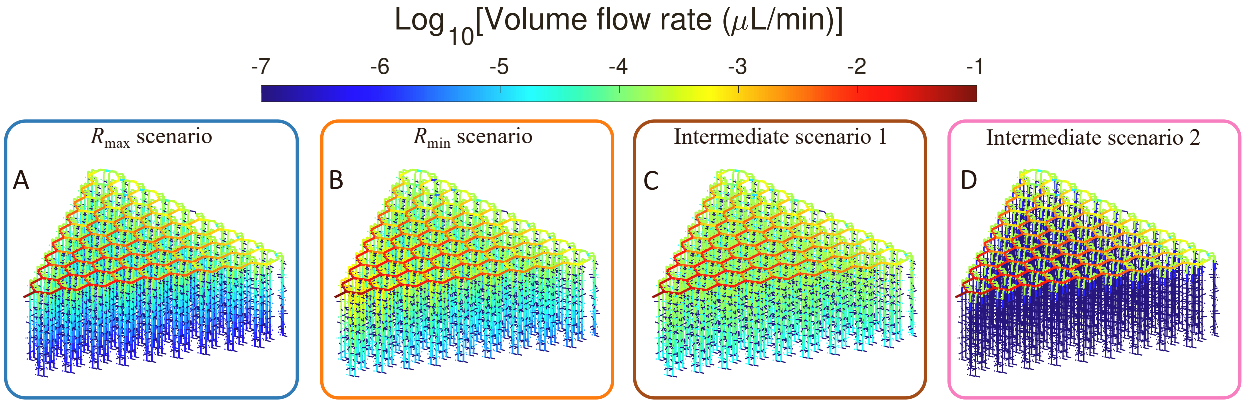

Figure S5: Spatial variation of volume flow rate, Related to Figure 3. Schematic diagrams of the network model with color indicating the volume flow rate. The scenarios are indicated at the top of each box; only the case of small precapillary PVSs is plotted ((A,D) $\Gamma_{\text{precap}} = 0.17$ and (B, C) $\Gamma_{\text{precap}} = 0.07$) which is virtually indistinguishable from the large precapillary PVS case.

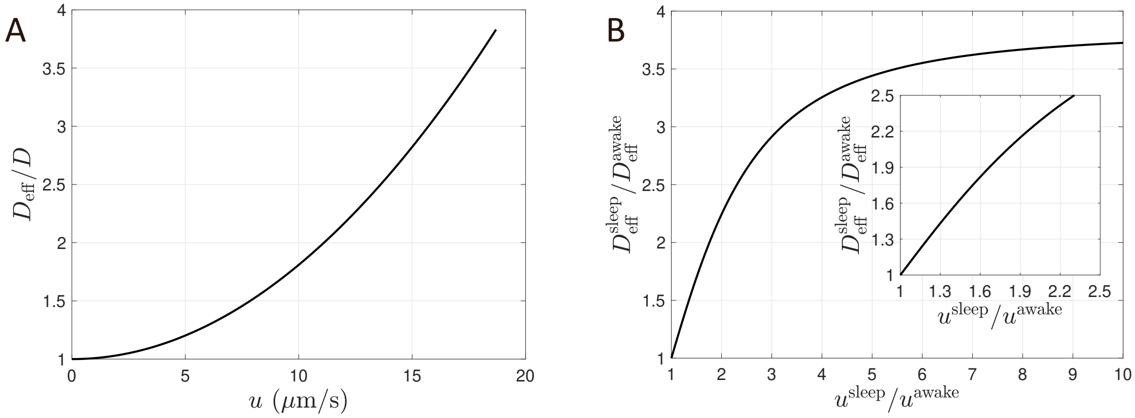

Figure S6: Sleep/awake dispersion coefficients for pial PVSs, Related to Figure 4. (A) The solute transport enhancement factor D_{eff}/D due to Taylor dispersion versus the mean flow speed u. Note that this calculation assumes the PVS shape is a concentric circular annulus with arterial diameter 46 μm and $\Gamma_{\text{pial}} = 1.4$. For $u = 18.7$ μm/s (Mestre et al 2018b) and $D = 1 \times 10^{-11}$ m^2/s, $D_{\text{eff}}/D = 3.8$. (B) The ratio of dispersion enhancement factors $D_{\text{eff}}^{\text{sleep}}/D_{\text{eff}}^{\text{awake}}$ as a function of the sleep-to-awake flow speed ratio $u^{\text{sleep}}/u^{\text{awake}}$, where $u^{\text{sleep}} = 18.7$ μm/s (Mestre et al 2018b). The inset shows a magnified view for small values.

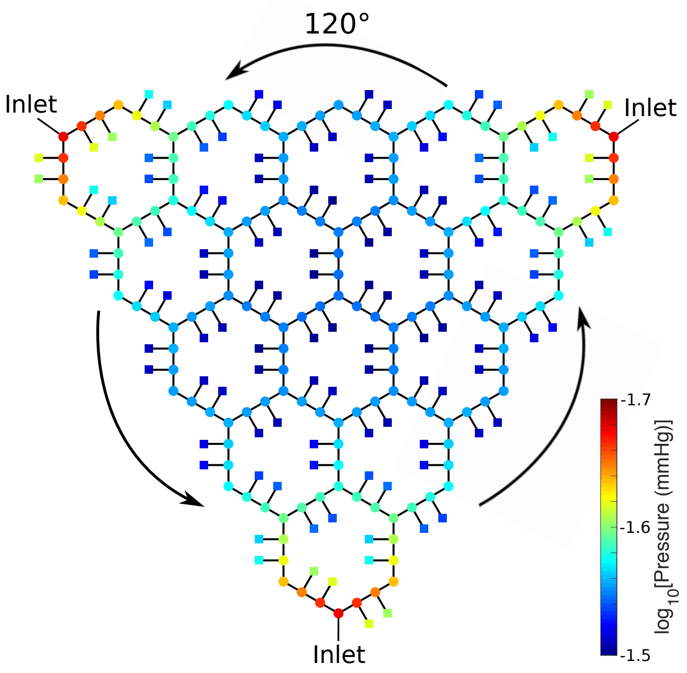

Figure S7: Additional model verification by testing rotational symmetry, Related to STAR Methods. By implementing a total of three inlets (which is non-physiological), the hydraulic network model exhibits a 120° rotational symmetry. By comparing a rotated network to the original network, we determined that the computed pressure at each node satisfies rotational symmetry to within 10^{-8}%. Note that only pial nodes are plotted for the sake of clarity.

Scenario	R_{pen}	R_{par}	R_{precap}	R_{offshoot}
R_{max} ($\Gamma_{\text{precap}} = 0.17$)	5.131×10^7	1.004×10^8	8.114×10^8	1.152×10^8
R_{max} ($\Gamma_{\text{precap}} = 0.36$)	5.131×10^7	1.004×10^8	3.832×10^8	1.116×10^8
R_{min} ($\Gamma_{\text{precap}} = 0.07$)	3.018×10^5	2.737×10^5	9.986×10^9	1.174×10^5
R_{min} ($\Gamma_{\text{precap}} = 0.36$)	3.018×10^5	2.737×10^5	8.320×10^7	1.173×10^5
Intermediate 1 ($\Gamma_{\text{precap}} = 0.07$)	3.018×10^5	1.004×10^8	9.986×10^9	9.169×10^6
Intermediate 1 ($\Gamma_{\text{precap}} = 0.36$)	3.018×10^5	1.004×10^8	8.320×10^7	4.271×10^6
Intermediate 2 ($\Gamma_{\text{precap}} = 0.17$)	5.131×10^7	2.737×10^5	8.114×10^8	6.868×10^7
Intermediate 2 ($\Gamma_{\text{precap}} = 0.36$)	5.131×10^7	2.737×10^5	3.832×10^8	6.868×10^7

Table S1: Hydraulic resistances for different circuit elements in each of the eight scenarios, Related to Figure 3. The last column (R_{offshoot}) corresponds to the entire lumped resistance for each pial offshoot, including all channels (penetrating and precapillary PVSs, parenchymal flow, and efflux) from the pial bifurcation leading to a penetrating PVS (i.e., points where the black channel bifurcates to a red channel in Fig. 1B) to ground). Units for all resistance values are mmHg·min/ml.
